# Efficacy and Safety of Proprotein Convertase Subtilisin/Kexin Type 9 Inhibitors as Adjuvant Treatments for Patients with Hypercholesterolemia Treated with Statin: A Systematic Review and Network Meta-analysis

**DOI:** 10.3389/fphar.2022.832614

**Published:** 2022-04-04

**Authors:** Yi-Ting Huang, Li-Ting Ho, Hsin-Yin Hsu, Yu-Kang Tu, Kuo-Liong Chien

**Affiliations:** ^1^ Institute of Epidemiology and Preventive Medicine, College of Public Health, National Taiwan University, Taipei, Taiwan; ^2^ Department of Internal Medicine, National Taiwan University Hospital and College of Medicine, Taipei, Taiwan; ^3^ Division of cardiology, internal medicine department, National Taiwan University Hospital, Taipei, Taiwan; ^4^ Department of Family Medicine, Taipei MacKay Memorial Hospital, Taipei, Taiwan; ^5^ Department of Medicine, MacKay Medical College, New Taipei City, Taiwan

**Keywords:** PCSK9 inhibitors, add-on therapy, low-density lipoprotein cholesterol, hypercholesterolemia, atherogenic apolipoproteins

## Abstract

**Background:** The proprotein convertase subtilisin/kexin type 9 (PCSK9) inhibitors are potent LDL-C lowering agents. However, few head-to-head studies evaluated the efficacy on the lowering in other atherogenic apolipoproteins and safety of PCSK9 inhibitors at different dosages as an add-on statins therapy in hypercholesterolemia patients.

**Methods:** This study is a systematic review and network meta-analysis of randomized control trials to compare the efficacy of lipid reduction and adverse events of PCSK9 inhibitors in statin-treated hypercholesterolemia patients. PubMed, EMBASE, and Cochrane Library databases were searched till April 20, 2021, for randomized controlled trials. Random-effect network meta-analyses were undertaken to compare the differences in the percent reduction in low-density lipoprotein cholesterol (LDL-C), apolipoprotein B (ApoB), and lipoprotein (a) [Lp(a)] levels and the risk of AEs among different PCSK9 inhibitors.

**Results:** A total of 22 articles with 42,786 patients were included. The lipid reductions in LDL-C, ApoB, and Lp(a) with add-on PCSK9 inhibitors vs. placebo in statin-treated patients across all trials were 50–63%, 43–52%, and 23–31%, respectively. Evolocumab 140 mg Q2W was ranked the best among all treatment strategies for lowering LDL-C, ApoB, and Lp(a) levels, and the treatment difference was 68.05% (95% confidence interval (CI), 62.43% to 73.67) in LDL-C reduction, 54.95% (95% CI, 49.55% to 60.35%) in ApoB reduction, and 34.25% (95% CI, 27.59% to 40.91%) in Lp(a) reduction compared with the placebo. No significant risk difference of adverse events between PCSK9 inhibitors and placebo was found.

**Conclusion:** PCSK9 inhibitors showed a significant effect on the reduction in LDL-C, ApoB, and Lp(a) levels in statin-treated patients. Evolocumab 140 mg Q2W showed significantly larger degrees of LDL-C, ApoB, and Lp(a) reduction.

## Introduction

Dyslipidemia, especially a high level of low-density lipoprotein cholesterol (LDL-C), has long been a critical risk factor in the development of cardiovascular disease (CVD) (Benn et al., 2007; Stamler and Neaton, 2008; Contois et al., 2009; Mellwig et al., 2017; Abdullah et al., 2018; Andersson et al., 2019; Robinson et al., 2020). A target-driven, lipid-lowering treatment is essential for CVD prevention. Besides LDL-C as the primary lipid target for prevention of CVD, atherogenic (apo) lipoproteins beyond LDL-C, such as apolipoprotein B (ApoB) and lipoprotein (a) [Lp(a)], may account for the residual cardiovascular risks (Hermans and Fruchart, 2010; Dhindsa et al., 2020).

For the population at higher cardiovascular risk, especially those with established CVD, intensive lipid-lowering has come to a consensus. However, failure to attain the lipid treatment target was observed despite evidence-based therapy with maximally tolerated statins (Fiévet and Staels, 2009; Hermans and Fruchart, 2010; Mach et al., 2020). Therefore, in very high–CVD risk patients, add-on statin therapy with other lipid-lowering treatments to reach the lipid level goal (Fiévet and Staels, 2009; Gupta, 2015; Lepor and Kereiakes, 2015), that is, LDL-C less than 70 mg/dl is recommended by the lipid-lowering guidelines (Grundy et al., 2019; Mach et al., 2020).

Proprotein convertase subtilisin/kexin type 9 (PCSK9) facilitates the degradation of the low-density lipoprotein receptor (LDL-R) and hinders the clearance of LDL-C (Everett et al., 2015; Lepor and Kereiakes, 2015; Lloyd-Jones et al., 2016; Grundy et al., 2019). Monoclonal antibodies inhibiting PCSK9 function and small interfering RNA reducing PCSK9 synthesis led to higher hepatic LDL-R expression and lower plasma LDL-C levels. PCSK9 inhibitors and small interfering RNA are recommended for high–CVD risk patients unable to achieve the lipid-lowering target by maximally tolerated oral therapies, including statins and/or ezetimibe (Kosmas et al., 2018; Ray et al., 2020; Macchi et al., 2021).

Two PCSK9 inhibitors, alirocumab and evolocumab, are approved for LDL-C reduction (Everett et al., 2015; Macchi et al., 2021). Clinical trials of both PCSK9 inhibitors demonstrated significant reduction of LDL-C and other atherogenic apolipoproteins, such as apolipoprotein B and Lp(a), which are attributable to the residual cardiovascular risk (Lepor and Kereiakes, 2015). Moreover, evidence has revealed that PCSK9 inhibitors lead to a lower risk of subsequent cardiovascular events by intensive LDL-C lowering (Myers et al., 2019; Sabatine, 2019). The ODYSSEY trials showed that alirocumab as an add-on statin therapy achieved significantly greater reduction in the LDL-C level than placebo (Bays et al., 2015; Cannon et al., 2015; Kastelein et al., 2015; Kereiakes et al., 2015; Robinson et al., 2015; Farnier et al., 2016; Ginsberg et al., 2016; Roth et al., 2016; Teramoto et al., 2016), ranging from 32 to 70 percent. Similarly, evolocumab as an add-on therapy attained around 46 to 72 percent greater reduction in the LDL-C level than placebo (Giugliano et al., 2012; Raal et al., 2012; Blom et al., 2014; Hirayama et al., 2014; Robinson et al., 2014; Raal et al., 2015a; Raal et al., 2015b; Kiyosue et al., 2016; Sabatine et al., 2017). In 2017, Schmidt et al. conducted a Cochrane systematic review and meta-analysis to evaluate the effect of PCSK9 inhibitors in reducing LDL-C and CVD risk, concluding that PCSK9 inhibitors reduced LDL-C and decreased CVD risk but may have increased the risk of any adverse events and led to little or no difference in mortality (Schmidt et al., 2017).

Inclisiran, a novel therapeutic agent, decreases PCSK9 hepatic synthesis by small interfering RNA (siRNA) (Kosmas et al., 2018). Inclisiran has been recently approved by the European Union since December 2020 for combination use with other lipid-lowering treatments or monotherapy to attain the lipid-lowering goal. ORION trials demonstrated that compared with placebo, inclisiran as an add-on statin therapy effectively reduced around 50% LDL-C level with no severe adverse reaction reported (Ray et al., 2017; Ray et al., 2020).

However, limited head-to-head studies compare the efficacy and safety of PCSK9 inhibitors to each other as add-on statin therapy. Most recent systematic reviews with meta-analyses have pooled PCSK9 inhibitors as a class (Li et al., 2015; Navarese et al., 2015; Zhang et al., 2015; Lipinski et al., 2016; Peng et al., 2016). Therefore, we conducted a systematic review and network meta-analysis to compare the efficacy of different PCSK9 inhibitors with different dosage as an add-on statin therapy in reducing the levels of LDL-C and lipoproteins e.g., ApoB and Lp(a), which are also important causal agents of atherosclerosis, and reducing cardiac events and the safety in adults with hyperlipidemia.

## Methods

### Search Strategy and Selection Criteria

Two investigators (Y-T Huang and L-T Ho) independently searched PubMed, Embase, Cochrane CENTRAL, Web of Science, and ClinicalTrials.gov, by applying the following keywords: “HMG-CoA reductase inhibitor*” or “Statin*” and “Proprotein convertase subtilisin*kexin type 9” or “Alirocumab” or “REGN727” or “SAR236553” ‘Praluent” or “Evolocumab” or “AMG 145” or “Repatha” or “Bococizumab” or “RN316” or “PF-04950615” or “Frovocimab” or “LY3015014” or “Inclisiran” or “ALN-PCSsc” or “RG7652” or “MPSK-3169A” or “Ebronucimab” or “AK102” or “JS002” or “Lerodalcibep” or “IBI306” or “CIVI007” from inception to April 20, 2021, without language restrictions. Detailed search strategies and the study protocol are provided in Supplementary Appendix S1. The study protocol was registered in the International Prospective Register of Systematic Reviews (CRD42017067529).

Studies to be included in our systematic review needed to fulfill the following criteria: 1) patients were randomly allocated to different treatments; 2) patients had one of the following conditions: LDL-C greater than 70 mg/dl, hypercholesterolemia, hyperlipidemia, mixed dyslipidemia, or high cardiovascular risk; 3) the study included comparisons of PCSK9 inhibitor therapies, with ezetimibe or placebo control; 4) the therapies should be add-on statin therapy; 5) the study reported changes in LDL-C, ApoB, or Lp(a); 6) the study was a phase 3 clinical trial. We excluded the bococizumab-related trials because the drug was discontinued for further development and was not approved for medical use.

### Data Extraction and Quality Assessment

**FIGURE 1:**
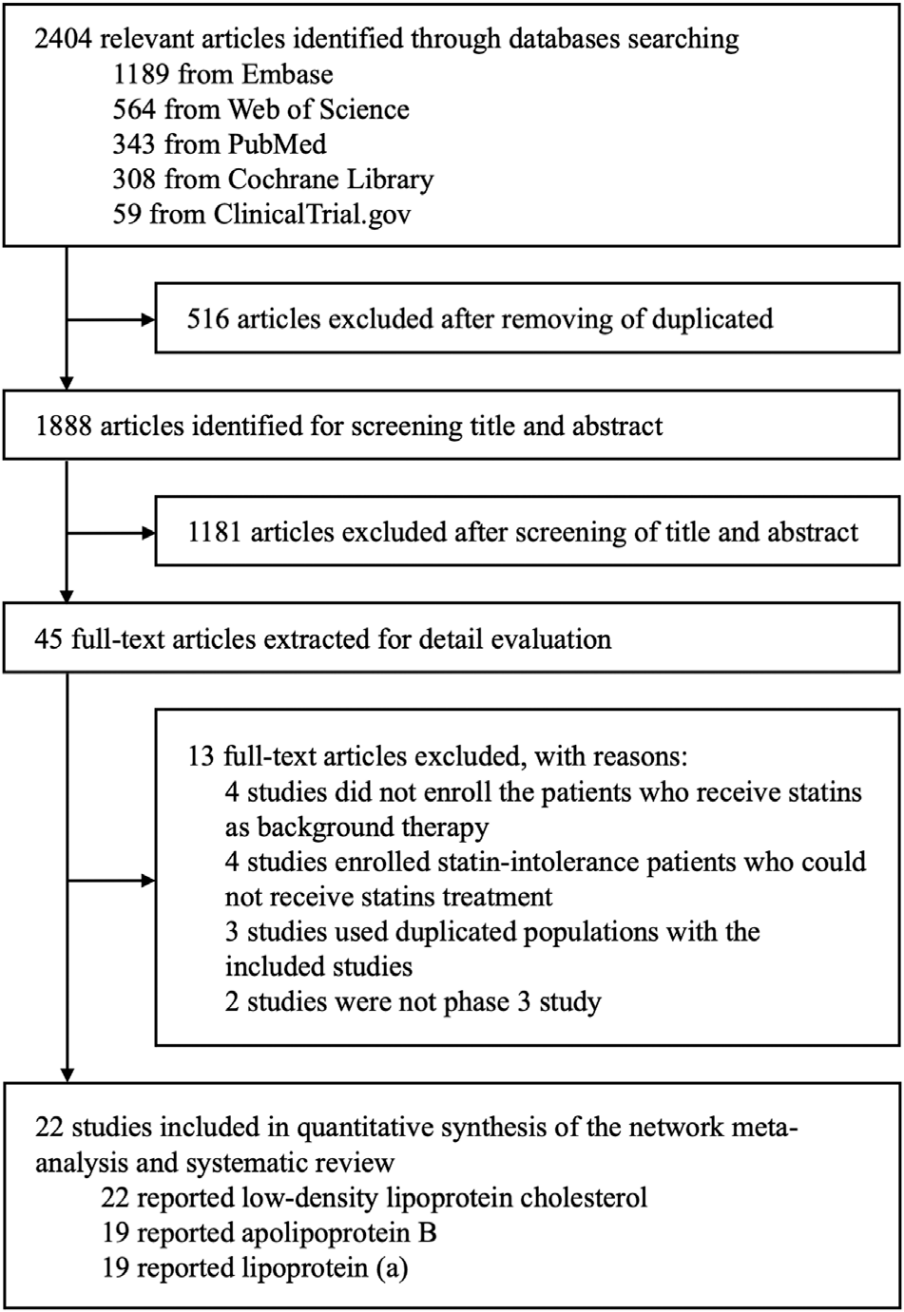
Flow chart of study identification and eligibility.

Two investigators (Y-T Huang and L-T Ho) independently reviewed full manuscripts of eligible studies. We used a structured database to ensure accuracy of data extraction (Figure 1). For a dose-ranging study, we included the doses: Alirocumab 75 mg or 150 mg biweekly (Q2W) and 300 mg monthly (QM); evolocumab 140 mg biweekly (Q2W) and 420 mg monthly (QM); inclisiran 300 mg with initial 3-month interval and every 6 months into the network meta-analysis. For efficacy analysis, the percentage changes in LDL-C, ApoB, and Lp(a) and the associated standard errors were extracted. For safety evaluation, the numbers of patients with the occurrence of adverse events (AEs), such as nasopharyngitis, injection-site reaction, or serious adverse events (SAEs), during the period from the initial injection to the end of study drug effect were extracted. When data required for our review were incomplete or lacked sufficient details, we contacted the original authors to request further information by email.

The same investigators independently assessed the study quality to evaluate the potential biases within the included studies. The studies were given a score of low, unclear, or high risk for selection bias, performance bias, detection bias, attrition bias, reporting bias, and other bias following the Cochrane Review Group’s Study Quality Guide, and the result of our evaluation was recorded using Review Manager software from the Cochrane Collaboration (Higgins and Green, 2011).

### Statistical Analysis

We conducted a network meta-analysis to compare the efficacy and safety among the PCSK9 inhibitors. The weighted mean differences in LDL-C, ApoB, and Lp(a) with the corresponding 95% CI were estimated for the efficacy of different PCSK9 inhibitors relative to placebo or ezetimibe. The odds ratio (OR) with the corresponding 95% CI was estimated for the incidence of AEs. In a frequentist setting, random-effects network meta-analysis combined direct and indirect evidence to provide a comprehensive evaluation of PCSK9 inhibitors. Within the network meta-analysis, we evaluated the consistency of evidence using three methods: 1) The design-by-treatment interaction model to evaluate the consistency in treatment effects between studies with different sets of treatments; 2) the loop inconsistency model to evaluate consistency in evidence with a closed loop; and 3) the node-splitting model to examine the difference between direct and indirect evidence for each pair of treatment (Salanti et al., 2008; Higgins et al., 2012; Tu, 2015; Yu-Kang, 2016). Moreover, we calculated the ranking probabilities for each treatment by undertaking 1,000 simulations to calculate the percentage of simulations for the performance of a treatment relative to other treatments in the network (Salanti et al., 2011). Heterogeneity was assessed using the Cochran Q test and the I^2^ statistic. I^2^ values of 25, 50, and 75% represented mild, moderate, and severe heterogeneity, respectively (Higgins et al., 2003). Small study or publication bias was examined by the funnel plot, Egger’s regression test, and Begg’s rank test (Egger et al., 1997). Sensitivity analyses were carried out to test the robustness of the study results. Statistical significance set at *p* < 0.05 was used for all statistical analyses. All analyses were conducted using R statistical software, version 3.6.1, with the package “netmeta”. Our analyses were in line with the recommendations of the Preferred Reporting Items for Systematic Reviews and Meta-Analyses (PRISMA) extension (Page et al., 2021).

## Results

A total of 2,404 articles were identified through database searching, and 1,888 articles remained after removing duplicates by screening the titles and abstracts. Among them, 1,843 articles that did not fulfill the inclusion criteria were excluded. The full texts of 45 potential articles were obtained for further assessment. Eventually, 22 articles with 42,786 patients were included in this systemic review and network meta-analysis (Figure 1).

### Basic Characteristics of Included Trials

Among 22 articles, 12 were alirocumab-based studies with 6,692 patients, eight were evolocumab-based studies with 32,434 patients, and two were inclisiran-based studies with 3,660 patients. The basic characteristics of the included studies are summarized in Table 1. Risk of bias was assessed in all included studies with seven domains of the Cochrane risk-of-bias assessment tool, which was at low risk of bias (Supplementary Figures S1, S2).

**TABLE 1:** Characteristics of included studies.

No	Year	Author	Trial	Duration, week(s)	Study population	Add-on therapy	Dosage of PCSK9 inhibitor (mg)	Sample size	Age, mean (SD)	Women, %	HP, %	DM, %	LDL-C, mean (SD)	ApoB, mean (SD)	Lp(a), median (SD)
Alirocumab															
1	2015	Bays H	ODYSSEY OPTIONS I	12/24	LDL-C ≥ 70 mg/dl with CVD or LDL-C ≥ 100 mg/dl with CVD risk factors	Alirocumab	75	57	62.2 (10.0)	42.1	77.2	57.9	103.9 (34.9)	90.0 (21.9)	24.0 (52.6)
						Ezetimibe	0	55	65.7 (9.0)	43.6	81.8	52.7	100.4 (29.5)	89.2 (22.6)	21.0 (27.4)
						Alirocumab	75	47	64.2 (10.4)	34	76.6	53.2	116.4 (37.4)	97.0 (25.5)	21.0 (44.4)
						Ezetimibe	0	47	63.9 (10.3)	23.4	78.7	34	98.9 (29.2)	83.3 (17.0)	32.0 (36.3)
2	2015/2017	Cannon CP/El Shahawy M	ODYSSEY COMBO II	24/52	LDL-C ≥ 70 mg/dl with CVD or LDL-C ≥ 100 mg/dl without CVD	Alirocumab	75	479	61.7 (9.4)	24.8	—	30.3	108.1 (34.7)	90.0 (20.0)	28.0 (44.8)
						Ezetimibe	0	241	61.3 (9.2)	29.5	—	31.5	104.2 (34.7)	90.0 (20.0)	22.4 (36.4)
3	2015	Kastelein JJP	ODYSSEY FH I, ODYSSEY FH II	24	LDL-C ≥ 70 mg/dl and TG ≤ 400 mg/dl with HeFH	Alirocumab	75	323	52.1 (12.9)	44.3	43	9.9	144.7 (52.1)	114.6 (30.7)	51.5 (2.8)
						Placebo	0	163	51.7 (12.3)	42.3	43.6	15.3	144.4 (37.0)	113.7 (28.4)	46.9 (4.0)
						Alirocumab	75	167	53.2 (12.9)	48.5	34.1	4.2	134.6 (37.5)	108.0 (27.7)	49.9 (5.4)
						Placebo	0	82	53.2 (12.5)	45.1	29.3	3.7	134.0 (26.3)	107.7 (23.9)	50.9 (6.6)
4	2015	Kereiakes DJ	ODYSSEY COMBO I	24	LDL-C ≥ 70 mg/dl with CVD or LDL-C ≥ 100 mg/dl with CHD risk	Alirocumab	75	209	63.0 (9.5)	37.3	—	45	100.2 (29.5)	90.8 (21.4)	31.0 (54.1)
						Placebo	0	107	63.0 (8.8)	28	—	39.3	106.0 (35.3)	91.4 (24.1)	38.0 (44.4)
5	2015	Robinson JG	ODYSSEY LONG TERM	24	LDL-C ≥ 70 mg/dl with HeFH or CHD	Alirocumab	150	1,530	60.4 (10.4)	36.7	—	34.9	122.7 (42.6)	101.9 (27.7)	22.2 (43.6)
						Placebo	0	780	60.6 (10.4)	39.8	—	33.9	121.9 (41.4)	101.1 (27.3)	20.9 (44.7)
6	2016	Farnier M	ODYSSEY OPTIONS II	12/24	LDL-C ≥ 70 mg/dl with CVD or LDL-C ≥ 100 mg/dl with CVD risk factors	Alirocumab	75	49	62.2 (11.1)	36.7	73.5	38.8	106.0 (29.1)	93.4 (22.6)	22.0 (48.9)
						Ezetimibe	0	48	60.4 (10.4)	45.8	68.8	47.9	94.7 (33.6)	89.0 (25.9)	38.5 (68.1)
						Alirocumab	75	54	57.9 (8.9)	48.1	74.1	33.3	114.1 (30.0)	92.7 (25.2)	49.5 (65.9)
						Ezetimibe	0	53	63.1 (10.2)	41.5	67.9	39.6	115.2 (48.4)	97.8 (20.4)	35.5 (45.2)
7	2016	Ginsberg HN	ODYSSEY HIGH FH	24	LDL-C ≥ 160 mg/dl with HeFH	Alirocumab	150	72	49.8 (14.2)	51.4	55.6	12.5	196.3 (57.9)	138.2 (32.0)	22.0 (31.1)
						Placebo	0	35	52.1 (11.2)	37.1	60	17.1	201.0 (43.4)	146.6 (28.3)	30.0 (23.0)
8	2016	Roth EM	ODYSSEY CHOICE I	24	LDL-C ≥70 mg/dl with moderate-to-very-high CVD risk or LDL-C ≥ 100 mg/d with moderate CVD risk	Alirocumab	75	78	60.7 (9.1)	34.6	—	28.2	118.0 (35.1)	99.6 (25.0)	28.0 (35.9)
						Alirocumab	300	312	61.6 (10.0)	39.1	—	30.8	115.4 (30.6)	96.6 (21.3)	27.0 (43.0)
						Placebo	0	157	61.6 (9.7)	35.7	—	31.8	115.8 (37.2)	96.0 (24.3)	25.5 (48.9)
9	2016	Teramoto T	ODYSSEY JAPAN	24	LDL-C ≥100 mg/dl with HeFH or Non-FH with high CAD risk or LDL-C ≥ 120 mg/dl	Alirocumab	75	144	60.3 (9.7)	41.7	—	72.9	142.9 (27.0)	110.0 (20.0)	16.8 (19.1)
						Placebo	0	72	61.8 (9.0)	34.7	—	59.7	142.9 (27.0)	110.0 (20.0)	14.7 (18.7)
10	2017	Leiter LA	ODYSSEY DM-INSULIN	24	LDL-C levels ≥70 mg/dl	Alirocumab	75	294	63.9 (8.9)	45.2	—	100	112.1 (34.3)	97.0 (24.7)	16.0 (37.0)
						Placebo	0	147	64.0 (9.4)	46.9	—	100	110.5 (37.4)	96.2 (26.8)	14.0 (24.4)
						Alirocumab	75	51	54.9 (10.1)	43.1	—	100	127.7 (58.1)	99.7 (35.6)	17.0 (16.3)
						Placebo	0	25	58.5 (7.8)	32	—	100	109.8 (31.4)	87.0 (21.0)	12.0 (24.4)
11	2018	Koh KK	ODYSSEY KT	24	LDL-C ≥ 70 mg/dl with a history of documented CVD, or LDL-C ≥ 100 mg/dl without such history	Placebo	0	102	60.1 (9.1)	20.6	—	37.3	99.3 (25.2)	85.6 (17.7)	24.5 (33.3)
						Alirocumab	75	97	61.2 (10.4)	14.4	—	33	97.0 (27.8)	81.7 (17.2)	23.0 (31.1)
12	2019	Han Y	ODYSSEY EAST	24	LDL-C ≥ 70 mg/dl with CVD or LDL-C ≥ 100 mg/dl without CVD	Alirocumab	75	407	58.8 (10.7)	22.6	63.4	29.7	110.7 (48.5)	94.7 (28.6)	28.0 (47.0)
						Ezetimibe	0	208	58.3 (11.2)	29.8	53.4	23.1	111.2 (49.8)	95.5 (30.5)	31.0 (50.0)
Evolocumab															
13	2014	Blom DJ	DESCARTES	52	LDL-C ≥ 75 mg/dl and TG ≤ 400 mg/dl	Placebo	0	129	57.0 (10.6)	21.3	39.5	7.8	98.4 (14.5)	82.6 (11.0)	12.1 (28.7)
						Evolocumab	420	254	57.2 (10.3)	57.1	42.9	6.7	101.3 (15.1)	84.0 (12.6)	12.1 (21.3)
						Placebo	0	73	58.4 (8.7)	54.8	56.2	19.2	96.2 (13.3)	83.3 (12.4)	21.7 (49.1)
						Evolocumab	420	145	57.8 (9.4)	47.6	57.9	13.1	94.6 (12.9)	83.3 (12.5)	30.8 (52.8)
						Placebo	0	63	55.9 (9.0)	47.6	60.3	25.4	119.8 (32.4)	100.3 (22.1)	26.3 (65.4)
						Evolocumab	420	126	54.2 (11.5)	44.4	54	19.8	116.8 (35.3)	95.5 (23.6)	27.9 (48.2)
14	2014	Robinson JG	LAPLACE-2	12	LDL-C ≥ 80 mg/dl with intensive statin and TG ≤ 400 mg/dl	Placebo	0	56	58.3 (10.5)	42.9	—	16.1	123.0 (46.6)	95.3 (26.0)	13.1 (23.0)
						Placebo	0	55	62.2 (10.4)	50.9	—	12.7	123.7 (47.9)	95.3 (29.6)	17.1 (28.1)
						Ezetimibe	0	56	61.0 (9.0)	51.8	—	10.7	126.8 (49.6)	101.3 (31.2)	15.4 (55.7)
						Ezetimibe	0	55	60.6 (9.2)	50.9	—	20	119.3 (28.1)	94.6 (20.4)	13.8 (47.8)
						Evolocumab	140	110	58.3 (8.4)	50.9	—	20.9	124.2 (43.4)	99.7 (26.4)	11.3 (34.6)
						Evolocumab	420	110	59.6 (11.1)	40	—	13.6	126.1 (50.4)	97.3 (28.9)	20.4 (48.8)
						Placebo	0	55	57.1 (9.9)	40	—	12.7	100.3 (36.2)	81.1 (22.1)	24.6 (50.0)
						Placebo	0	55	58.8 (11.5)	43.6	—	18.2	94.7 (31.9)	80.1 (21.4)	20.8 (42.9)
						Ezetimibe	0	56	60.5 (10.2)	42.9	—	17.9	98.7 (34.0)	85.3 (23.1)	10.4 (29.6)
						Ezetimibe	0	54	61.1 (8.9)	51.9	—	31.5	92.3 (19.3)	78.7 (16.7)	25.6 (55.5)
						Evolocumab	140	109	59.7 (10.2)	39.4	—	14.7	94.2 (34.8)	79.9 (25.1)	13.3 (38.3)
						Evolocumab	420	110	60.1 (10.2)	43.6	—	16.4	93.8 (32.3)	77.9 (21.5)	10.2 (26.3)
						Placebo	0	56	61.9 (9.7)	57.1	—	17.9	110.3 (28.0)	91.6 (18.4)	14.2 (46.3)
						Placebo	0	55	61.5 (10.3)	50.9	—	20	108.6 (30.9)	89.8 (20.7)	14.6 (44.0)
						Evolocumab	140	112	59.7 (9.2)	40.2	—	17.9	114.9 (34.9)	94.2 (24.0)	15.8 (47.5)
						Evolocumab	420	115	61.5 (9.6)	51.3	—	13	123.7 (48.5)	96.5 (27.5)	13.3 (50.3)
						Placebo	0	58	61.2 (9.1)	60.3	—	5.2	115.6 (39.8)	93.1 (27.3)	14.2 (46.3)
						Placebo	0	57	59.6 (9.2)	47.4	—	15.8	119.9 (39.1)	95.9 (25.2)	14.6 (44.0)
						Evolocumab	140	113	58.9 (11.2)	45.1	—	23	118.7 (40.9)	95.4 (27.0)	15.8 (47.5)
						Evolocumab	420	115	59.3 (10.5)	44.3	—	10.4	122.9 (42.0)	97.2 (26.9)	13.3 (50.3)
						Placebo	0	56	60.2 (8.7)	37.5	—	3.6	77.4 (20.9)	71.0 (16.6)	11.9 (50.6)
						Placebo	0	55	58.1 (11.4)	47.3	—	10.9	102.9 (49.3)	84.8 (29.7)	13.8 (42.3)
						Evolocumab	140	111	59.5 (9.2)	38.7	—	16.2	88.5 (31.5)	77.4 (22.3)	17.1 (53.4)
						Evolocumab	420	112	59.6 (9.0)	46.4	—	10.7	88.5 (31.3)	78.7 (23.1)	20.6 (53.5)
15	2015	Raal FJ	TESLA Part B	12	HoFH	Placebo	0	16	32 (14)	50	—	—	335.9 (146.7)	210.0 (80.0)	53.3 (37.3)
						Evolocumab	420	33	30 (12)	48	—	—	355.2 (135.1)	210.0 (70.0)	31.7 (36.7)
16	2015	Raal FJ	RUTHERFORD-2	12	HeFH	Placebo	0	54	51.1 (14.2)	46	—	—	150.6 (34.7)	110.0 (30.0)	18.3 (25.0)
						Evolocumab	140	110	52.6 (12.3)	40	—	—	154.4 (50.2)	120.0 (30.0)	32.3 (54.5)
						Placebo	0	55	46.8 (12.1)	44	-—	—	150.6 (42.5)	110.0 (20.0)	36.3 (56.5)
						Evolocumab	420	110	51.9 (12.0)	42	—	—	154.4 (42.5)	110.0 (30.0)	25.4 (54.6)
17	2016	Kiyosue A	YUKAWA-2	12	LDL-C ≥ 100 mg/dl and TG ≤ 400 mg/dl	Placebo	0	202	61.0 (10.0)	39	72	51	103.0 (28.0)	92.0 (20.0)	12.9 (11.7)
						Evolocumab	140/420	202	62.0 (11.0)	40	75	47	109.0 (35.0)	96.0 (25.0)	14.2 (14.5)
18	2017	Sabatine MS	FOURIER	12	LDL-C ≥ 70 mg/dl with atherosclerotic vascular disease or LDL-C ≥ 100 mg/dl without atherosclerotic vascular disease	Evolocumab	140/420	13,784	32.5 (9.1)	24.6	80.1	36.7	92 (21.5)	—	15.4 (47.2)
						Placebo	0	13,780	62.5 (8.9)	24.5	80.1	36.5	92 (21.5)	—	15.4 (46.6)
19	2019	Lorenzatti AJ	BERSON	12	LDL-C ≥100 mg/dl with DM	Evolocumab	140/420	657	62 (34.8)	55.9	72.8	100	92.8 (34.8)	—	28.9 (39.3)
						Placebo	0	324	62 (33.3)	60.2	73.8	100	92.8 (30.9)	—	28.9 (39.0)
20	2019	Rosenson RS	BANTING	12	LDL-C ≥70 mg/dl and non-HDL-C ≥100 mg/dl with DM and CVD, or LDL-C ≥100 mg/dl and non-HDL-C ≥130 mg/dl with DM and without CVD	Evolocumab	420	280	62.5 (8.5)	42.9	88.2	100	108.7 (30.9)	97.0 (23.0)	36.7 (46.5)
						Placebo	0	141	62.2 (8.4)	46.1	84.4	100	110.6 (32.9)	98.0 (22.0)	41.4 (51.2)
Inclisiran															
21	2020	Raal FJ	ORION-9	78	LDL-C ≥100 mg/dL with FH	Inclisiran	300	242	56 (11.9)	53.7	42.1	8.3	151.4 (50.4)	123.8 (33.2)	23.8 (65.8)
						Placebo	0	240	56 (13.3)	52.1	42.1	11.7	154.7 (58.0)	124.5 (34.8)	22.5 (68.8)
22	2020	Raal KK	ORION-10	78	LDL-C ≥70 mg/dl with ASCVD	Inclisiran	300	781	66.4 (8.9)	31.5	91.4	47.5	104.5 (39.6)	94.1 (25.6)	23.8 (72.1)
						Placebo	0	780	65.7 (8.9)	29.7	89.9	42.4	104.8 (37.0)	94.6 (25.1)	23.3 (70.4)
			ORION-11		LDL-C ≥70 mg/dl with ASCVD factors	Inclisiran	300	810	64.8 (8.3)	28.5	79	36.5	107.2 (41.8)	97.1 (28.0)	17.5 (66.7)
						Placebo	0	807	84.8 (8.7)	28	81.9	33.7	103.7 (36.4)	95.1 (5.2)	14.6 (67.9)

HP: hypertension; DM: type 2 diabetes mellitus; LDL-C: low-density lipoprotein cholesterol; ApoB: apolipoprotein B; Lp(a): lipoprotein(a).

### Efficacy Endpoints

**FIGURE 2:**
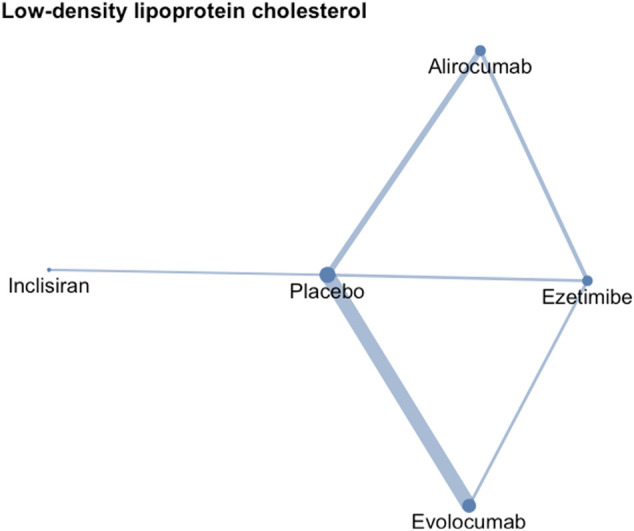
Network geometry of low-density lipoprotein cholesterol. The sizes of treatment nodes reflect the number of patients randomly assigned to each treatment. The thicknesses of edges represent the number of studies underlying each comparison.

The network meta-analysis contained eight treatments, including PCSK9 inhibitors at different dosage, including alirocumab 75 mg/150 mg Q2W, 300 mg QM, and evolocumab 140 mg every Q2W, 420 mg every QM, and inclisiran 300 mg with an initial 3-month interval and every six months versus either ezetimibe or placebo (Figure 2).

### Low-Density Lipoprotein Cholesterol

**FIGURE 3:**
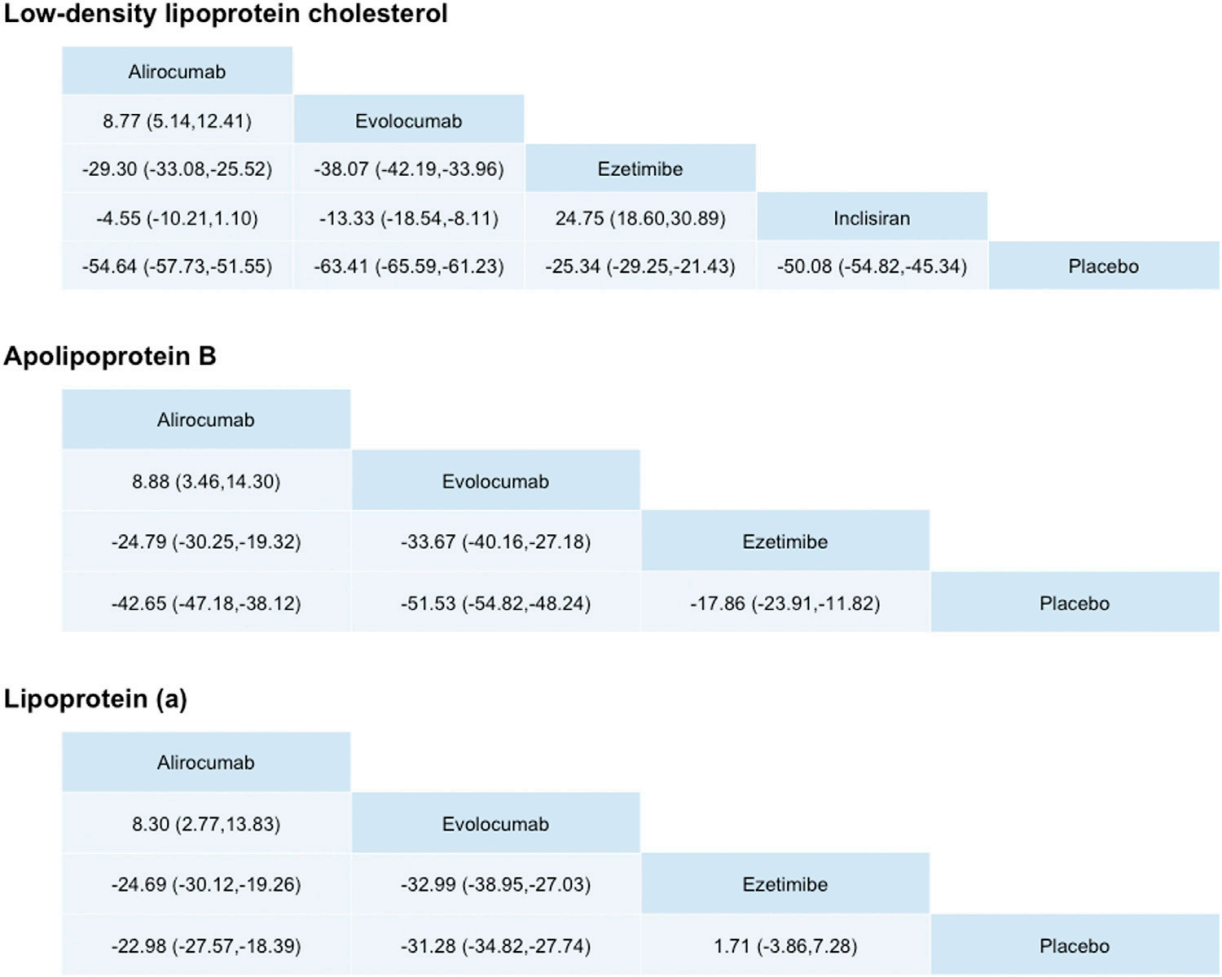
Differences in percentage changes in low-density lipoprotein cholesterol, ApoB, and lipoprotein (a) obtained by network meta-analysis. Comparisons should be read from left to right. A positive value favors the column treatment.

**FIGURE 4:**
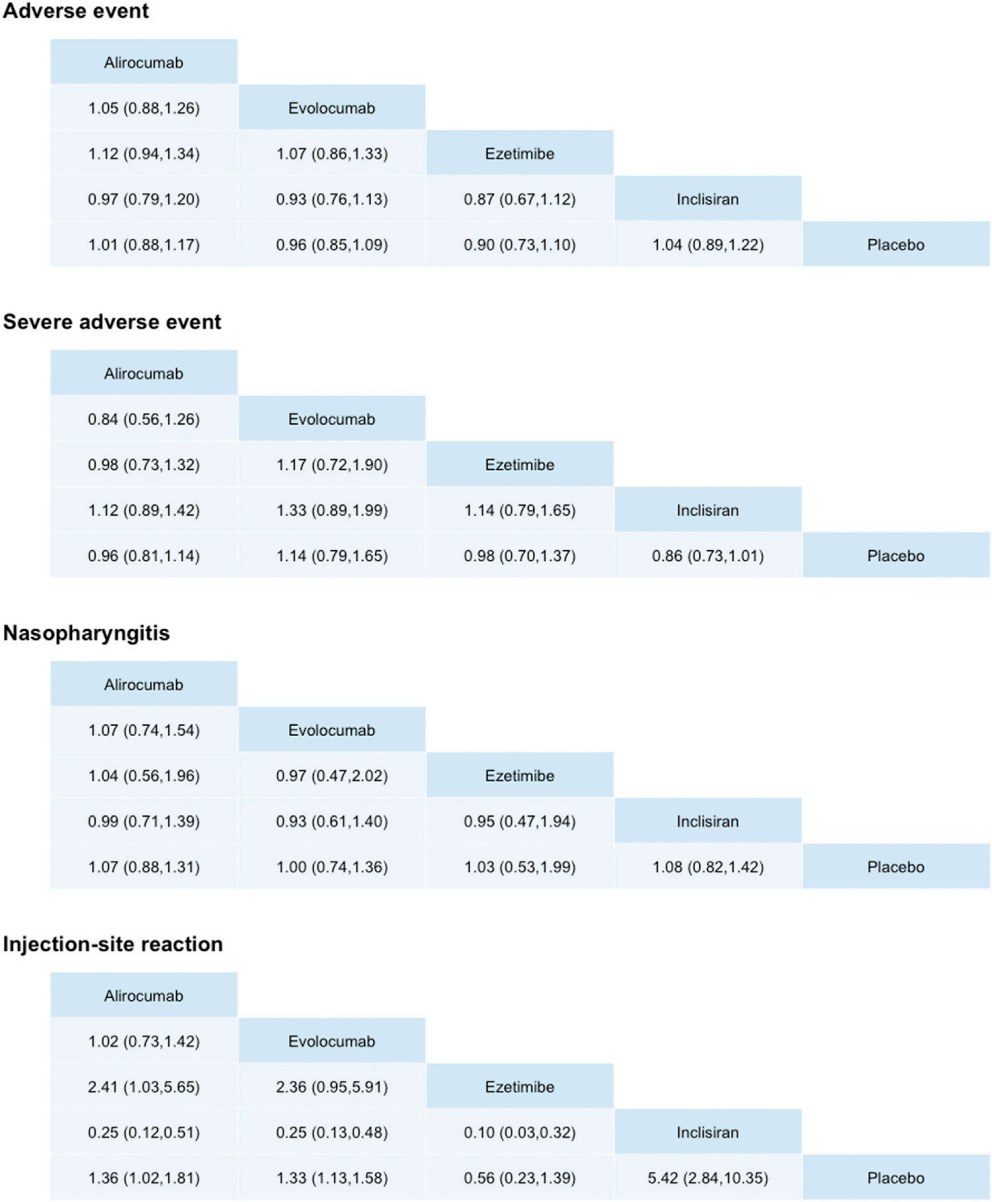
Odds ratios of adverse events, nasopharyngitis, injection-site reaction, and serious adverse events obtained by network meta-analysis. Comparisons should be read from left to right. An odds ratio smaller than 1 favors the column treatment.

The upper part of Figure 3 and Figure 4 present the results of network meta-analyses for LDL-C reduction. Among three PCSK9 inhibitors, evolocumab had greater LDL-C reduction than alirocumab or inclisiran. Evolocumab 140 mg Q2W was ranked as the best among all treatment strategies for lowering LDL-C levels, and the treatment difference was 68.05% (95% CI: 62.43–73.67%) compared with placebo. Evolocumab 140 mg Q2W had greater LDL-C reduction than evolocumab 420 mg QM [58.01% (53.65%, 62.37%)], alirocumab 75/150 mg Q2W [53.72% (49.75%, 57.70%)], and inclisiran 300 mg [47.90% (35.54%, 60.26%)] (Supplementary Figure S5). However, evolocumab 420 mg QM had similar LDL-C level reduction effects compared with those of alirocumab 300 mg QM [59.70% (48.01%, 71.38%)]. Compared with ezetimibe, all PCSK9 inhibitors had significant effects on LDL-C reduction. Nevertheless, as an add-on lipid-lowering therapy, ezetimibe still significantly reduced LDL-C levels compared with placebo.

### Apolipoprotein B

The middle part of Figure 3 presents the results of network meta-analyses for ApoB reduction. Among three PCSK9 inhibitors, all significantly reduced ApoB levels compared with placebo. As the trend of LDL-C reduction, evolocumab had greater ApoB reduction than alirocumab. Evolocumab 140 mg Q2W was ranked the best among all treatment strategies (inclisiran was excluded because no ApoB lowering data were available for lowering ApoB levels, and the treatment difference was 54.95% [49.55% and 60.35%) compared with placebo, but similar compared with alirocumab 300 mg QM [49.41% (38.22%, 60.60%)] (Supplementary Figure S5).

Evolocumab 140 mg Q2W had greater ApoB reduction than evolocumab 420 mg QM [46.22% (46.06%, 50.37%)] and alirocumab 75/150 mg Q2W [42.42% (38.54%, 46.29%)]. Compared with ezetimibe, different dosages of evolocumab and alirocumab had significant effects on ApoB reduction. Nevertheless, as an add-on lipid-lowering therapy, ezetimibe still significantly reduced LDL-C levels compared with placebo.

### Lipoprotein (a)

The lower part of Figure 3 presents the results of network meta-analyses for Lp(a) reduction. Among three PCSK9 inhibitors, all significantly reduced Lp(a) levels compared with placebo. However, the effects of each PCSK-9 on Lp(a) were different from the effects on LDL-C or ApoB, although evolocumab 140 mg Q2W remained the best ranking among all treatment strategies for lowering Lp(a) levels [34.25% (27.59%, 40.91%)] compared with placebo. However, the differences in Lp(a) reduction were not significantly different between evolocumab and alirocumab at different dosages.

### Safety Endpoints

Regarding the safety of PCSK9 inhibitors, no significant risk of AEs, SAEs, or nasopharyngitis were noted (Contois et al., 2009). Only inclisiran increased the risk of injection-site reaction. This study suggests that PCSK9 inhibitors were safe with tolerable side effects as an adjuvant lipid-lowering therapy.

### Bias Assessment, Inconsistency Assessment, and Sensitivity Analyses

Although Egger’s test implied that there might be publication bias for percentage change in ApoB (Supplementary Figures S19, S20), the funnel plot showed that the possible source of asymmetry might be from larger studies, which might be resulted from heterogeneity. For the assessment of publication bias for AEs from PCSK9 inhibitors, no significant publication bias was found. For the inconsistency assessment (Supplementary Table S1), overall inconsistency between the designs-interaction random-effect model was not found. Sensitivity analyses were performed using an alternative meta-analysis model, that is, the fixed-effect model, and the results remained consistent with our main results (Supplementary Figures S26, S27).

## Discussion

The present study evaluated the efficacy and safety of different PCSK9 inhibitors as adjuvant therapies in statin-treated hypercholesterolemic patients. The statin used in the included trials were maximally tolerated statin therapy; most of the doses the trials applied were moderate-to-high intensity statin dose, that is, atorvastatin 20, 40, or 80 mg once a day; rosuvastatin 20 or 40 mg once a day; and simvastatin 40 mg or 80 mg once a day. Moderate-to-high intensity statin therapy causes 30 to 50% LDL-C reduction (Oesterle et al., 2017). This study revealed that statin add-on PCSK9 inhibitors, including evolocumab, alirocumab, and inclisiran vs. placebo or ezetimibe, significantly reduced the levels of LDL-C, ApoB, and Lp(a). Among the PCSK9 inhibitors, evolocumab 140 mg Q2W was found to be more superior in atherogenic lipid reduction, including LDL-C, ApoB, and Lp(a), than the others except for alirocumab 300 mg QM. PCSK9 inhibitors have similar side effects other than higher injection-site reaction caused by inclisiran. The approving risk-benefit results of evolocumab, alirocumab, and inclisiran in lipid lowering was consistent with previous literature (Strilchuk et al., 2019). However, the results of PCSK9 inhibitor benefit–risk ratio still need to be interpreted cautiously due to the AE of PCSK9 inhibitors being such rare events that the statistical power to detect the difference among studies may be relatively insufficient.

Previous meta-analyses found that compared with non–anti-PCSK9 treatment, anti-PCSK9 treatment noticeably reduced lipid profiles, and the incidence of AEs did not increase. These traditional meta-analyses combined all PCSK9 treatments into a single group of anti-PCSK9 treatments, so it did not provide information about the potential differences in efficacy and safety between various PCSK9 treatments (Li et al., 2015; Navarese et al., 2015; Zhang et al., 2015). Our study found significant differences in treatment efficacy among different PCSK9 inhibitors, and evolocumab appeared to be the best ranking PCSK9 inhibitor in reducing atherogenic lipid level, including LDL-C, ApoB, and Lp(a). In 2017, Toth et al. conducted a systematic review and meta-analysis, revealing that PCSK9 inhibitors as an add-on therapy significantly reduce LDL-C and demonstrating that evolocumab vs. alirocumab had larger reduction in the LDL-C level, which are consistent with our study results (Toth et al., 2017). However, the current study further included the trials of inclisiran to compare the lipid-lowering ability among the previous two PCSK9 inhibitors and the novel PCSK9-inhibiting agent.

The probable biological mechanism to explain our study results was that PCSK9 inhibitor vs. statin provides a further LDL-C level lowering by interfering in PCSK9 function. Evolocumab and alirocumab are human monoclonal antibodies that target PCSK9 approved for LDL-C reduction (Everett et al., 2015; Macchi et al., 2021). Inclisiran, a novel therapeutic agent, decreases PCSK9 hepatic synthesis by small interfering RNA (siRNA) (Ray et al., 2017; Kosmas et al., 2018). The monoclonal antibodies inhibiting PCSK9 function and small interfering RNA reducing PCSK9 synthesis led to higher hepatic LDL-R expression and lower plasma LDL-C levels. The LDL-C level has long been the primary target for cardiovascular risk prevention; thus, further LDL-C lowering may be beneficial for the further reduction of cardiovascular risk. Besides LDL-C lowering, substantial ApoB and Lp(a) lowering caused by PCSK9 inhibitors in statin-treated hypercholesteremia patients may contribute to additional reduction of residual cardiovascular risk (Sacks, 2006; Ridker et al., 2008; Lieb et al., 2018). The discrepancy of the degree of LDL-C and ApoB reduction may be due to the different physiologic roles in lipid metabolism. LDL-C represents the cholesterol mass of LDL particles, while ApoB reflects the total number of LDL, VLDL, and other atherogenic lipoprotein particles due to each of these lipoproteins being with one ApoB molecule. PCSK9 inhibitors keep LDL receptors from degradation to increase LDL-C uptake into cells for metabolism. However, not only LDL particles but also other atherogenic lipoproteins include ApoB, thus resulting in the dissociation between LDL-C and ApoB reduction trend (Sacks, 2006).

The latest 2017 update of ESC/EAS Task Force on practical clinical guidance for PCSK9 inhibitors (Landmesser et al., 2018) suggested that in atherosclerotic cardiovascular disease (ASCVD) patients with substantially elevated LDL-C levels, a PCSK9 inhibitor should be considered despite maximally tolerated statin with or without ezetimibe therapy or inability to tolerate appropriate doses of at least three statins, especially if there are additional indications of increased cardiovascular risk. Our study results provided robust evidence about the potent atherogenic lipid-lowering ability and safety of the PCSK9 inhibitors as adjuvant lipid-lowering treatments. In addition, this study revealed that evolocumab had greater lipid reduction than alirocumab or inclisiran. Moreover, evolocumab Q2W may be the best ranking choice in lipid lowering among all PCSK9 inhibitors at different dosages. However, mentioned knowledge gaps in the clinical guideline were noted concerning the application of the PCSK9 inhibitors, including interindividual variability, long-term efficacy, and especially long-term safety, thus further longitudinal studies are still warranted (Landmesser et al., 2018; Nelson et al., 2019; Kosmas et al., 2020).

Some limitations in the present study must be noted. First, we included PCSK9 inhibitors as adjunctive therapies studies with incomplete information about the background statin therapy. The impact of different statin dosages on the reduction in lipids was neglected in the present study. Second, since PCSK9 inhibitors are novel agents, evidence from randomized control trials is just emerging. For instance, our literature search only found two articles on inclisiran, and therefore, the confidence intervals of estimates on the efficacy and safety were relatively unstable, especially for the indirect estimation. Third, since the presence of publication bias was detected in some scenarios, the trim-and-fill method should be applied to assess how the summary estimate changes when these potentially missing studies are taken into account. However, to the best of our knowledge, the trim-and-fill method has still not been developed for conducting the network meta-analysis, whereas it could only adapt to the traditional meta-analysis. Finally, the R package we used cannot deal with trials with more than two treatments to perform the meta-regression for exploring an effect modifier. Further development of the package in R statistical software for meta-regression in network meta-analysis may be warranted.

## Conclusion

In this systematic review and network meta-analysis, PCSK9 inhibitors, as adjuvant treatment in statin-treated hypercholesterolemia patients, were associated with greater reduction in atherogenic lipid level, including LDL-C, ApoB, and Lp(a). Among PCSK9 inhibitors, evolocumab 140 mg Q2W showed significantly larger degrees of LDL-C, ApoB, and Lp(a) reduction than alirocumab 300 mg QM. No significant risk difference of AEs was found between PCSK9 inhibitors and placebo, except the higher injection-site reaction noted in inclisiran use.

## Data Availability

The original contributions presented in the study are included in the article/Supplementary Material; further inquiries can be directed to the corresponding authors.

## References

[B1] Andersson C. Johnson A. D. Benjamin E. J. Levy D. Vasan R. S. (2019). 70-year Legacy of the Framingham Heart Study. Nat. Rev. Cardiol. 16 (11), 687–698. 10.1038/s41569-019-0202-5 31065045

[B2] Bays H. Gaudet D. Weiss R. Ruiz J. L. Watts G. F. Gouni-Berthold I. (2015). Alirocumab as Add-On to Atorvastatin versus Other Lipid Treatment Strategies: ODYSSEY OPTIONS I Randomized Trial. J. Clin. Endocrinol. Metab. 100 (8), 3140–3148. 10.1210/jc.2015-1520 26030325 PMC4524987

[B3] Benn M. Nordestgaard B. G. Jensen G. B. Tybjaerg-Hansen A. (2007). Improving Prediction of Ischemic Cardiovascular Disease in the General Population Using Apolipoprotein B: the Copenhagen City Heart Study. Arterioscler Thromb. Vasc. Biol. 27 (3), 661–670. 10.1161/01.ATV.0000255580.73689.8e 17170368

[B4] Blom D. J. Hala T. Bolognese M. Lillestol M. J. Toth P. D. Burgess L. (2014). A 52-week Placebo-Controlled Trial of Evolocumab in Hyperlipidemia. N. Engl. J. Med. 370 (19), 1809–1819. 10.1056/NEJMoa1316222 24678979

[B5] Cannon C. P. Cariou B. Blom D. McKenney J. M. Lorenzato C. Pordy R. (2015). Efficacy and Safety of Alirocumab in High Cardiovascular Risk Patients with Inadequately Controlled Hypercholesterolaemia on Maximally Tolerated Doses of Statins: the ODYSSEY COMBO II Randomized Controlled Trial. Eur. Heart J. 36 (19), 1186–1194. 10.1093/eurheartj/ehv028 25687353 PMC4430683

[B6] Contois J. H. McConnell J. P. Sethi A. A. Csako G. Devaraj S. Hoefner D. M. (2009). Apolipoprotein B and Cardiovascular Disease Risk: Position Statement from the AACC Lipoproteins and Vascular Diseases Division Working Group on Best Practices. Clin. Chem. 55 (3), 407–419. 10.1373/clinchem.2008.118356 19168552

[B7] Dhindsa D. S. Sandesara P. B. Shapiro M. D. Wong N. D. (2020). The Evolving Understanding and Approach to Residual Cardiovascular Risk Management. Front. Cardiovasc. Med. 7, 88. 10.3389/fcvm.2020.00088 32478100 PMC7237700

[B8] Egger M. Davey Smith G. Schneider M. Minder C. (1997). Bias in Meta-Analysis Detected by a Simple, Graphical Test. BMJ 315 (7109), 629–634. 10.1136/bmj.315.7109.629 9310563 PMC2127453

[B9] Everett B. M. Smith R. J. Hiatt W. R. (2015). Reducing LDL with PCSK9 Inhibitors--The Clinical Benefit of Lipid Drugs. N. Engl. J. Med. 373 (17), 1588–1591. 10.1056/NEJMp1508120 26444323

[B10] Farnier M. Jones P. Severance R. Averna M. Steinhagen-Thiessen E. Colhoun H. M. (2016). Efficacy and Safety of Adding Alirocumab to Rosuvastatin versus Adding Ezetimibe or Doubling the Rosuvastatin Dose in High Cardiovascular-Risk Patients: the ODYSSEY OPTIONS II Randomized Trial. Atherosclerosis 244, 138–146. 10.1016/j.atherosclerosis.2015.11.010 26638010

[B11] Fiévet C. Staels B. (2009). Combination Therapy of Statins and Fibrates in the Management of Cardiovascular Risk. Curr. Opin. Lipidol. 20 (6), 505–511. 10.1097/MOL.0b013e328332e9ef 19829109 PMC2980504

[B12] Ginsberg H. N. Rader D. J. Raal F. J. Guyton J. R. Baccara-Dinet M. T. Lorenzato C. (2016). Efficacy and Safety of Alirocumab in Patients with Heterozygous Familial Hypercholesterolemia and LDL-C of 160 Mg/dl or Higher. Cardiovasc. Drugs Ther. 30 (5), 473–483. 10.1007/s10557-016-6685-y 27618825 PMC5055560

[B13] Giugliano R. P. Desai N. R. Kohli P. Rogers W. J. Somaratne R. Huang F. (2012). Efficacy, Safety, and Tolerability of a Monoclonal Antibody to Proprotein Convertase Subtilisin/kexin Type 9 in Combination with a Statin in Patients with Hypercholesterolaemia (LAPLACE-TIMI 57): a Randomised, Placebo-Controlled, Dose-Ranging, Phase 2 Study. Lancet 380 (9858), 2007–2017. 10.1016/s0140-6736(12)61770-x 23141813 PMC4347805

[B14] Grundy S. M. Stone N. J. Bailey A. L. Beam C. Birtcher K. K. Blumenthal R. S. (2019). 2018 AHA/ACC/AACVPR/AAPA/ABC/ACPM/ADA/AGS/APhA/ASPC/NLA/PCNA Guideline on the Management of Blood Cholesterol: Executive Summary: A Report of the American College of Cardiology/American Heart Association Task Force on Clinical Practice Guidelines. J. Am. Coll. Cardiol. 73 (24), 3168–3209. 10.1016/j.jacc.2018.11.002 30423391

[B15] Gupta S. (2015). LDL Cholesterol, Statins and PCSK 9 Inhibitors. Indian Heart J. 67 (5), 419–424. 10.1016/j.ihj.2015.05.020 26432726 PMC4593843

[B16] Higgins J. P: Cochrane Handbook for Systematic Reviews of Interventions Version 5.1.0. In The Cochrane Collaboration. Green S.. Chichester: John Wiley and Sons. Available at: training.cochrane.org/handbook/archive/v5.1/ . (Accessed March 11), (2011).

[B17] Hermans M. P. Fruchart J.-C. (2010). Reducing Residual Vascular Risk in Patients with Atherogenic Dyslipidemia: where Do We Go from Here? Clin. Lipidol. 5 (6), 811–826. 10.2217/clp.10.65

[B18] Higgins J. P. Jackson D. Barrett J. K. Lu G. Ades A. E. White I. R. (2012). Consistency and Inconsistency in Network Meta-Analysis: Concepts and Models for Multi-Arm Studies. Res. Synth. Methods 3 (2), 98–110. 10.1002/jrsm.1044 26062084 PMC4433772

[B19] Higgins J. P. Thompson S. G. Deeks J. J. Altman D. G. (2003). Measuring Inconsistency in Meta-Analyses. BMJ 327 (7414), 557–560. 10.1136/bmj.327.7414.557 12958120 PMC192859

[B20] Hirayama A. Honarpour N. Yoshida M. Yamashita S. Huang F. Wasserman S. M. (2014). Effects of Evolocumab (AMG 145), a Monoclonal Antibody to PCSK9, in Hypercholesterolemic, Statin-Treated Japanese Patients at High Cardiovascular Risk-Pprimary Results from the Phase 2 YUKAWA Study. Circ. J. 78 (5), 1073–1082. 10.1253/circj.CJ-14-0130 24662398

[B21] Kastelein J. J. Ginsberg H. N. Langslet G. Hovingh G. K. Ceska R. Dufour R. (2015). ODYSSEY FH I and FH II: 78 Week Results with Alirocumab Treatment in 735 Patients with Heterozygous Familial Hypercholesterolaemia. Eur. Heart J. 36 (43), 2996–3003. 10.1093/eurheartj/ehv370 26330422 PMC4644253

[B22] Kereiakes D. J. Robinson J. G. Cannon C. P. Lorenzato C. Pordy R. Chaudhari U. (2015). Efficacy and Safety of the Proprotein Convertase Subtilisin/kexin Type 9 Inhibitor Alirocumab Among High Cardiovascular Risk Patients on Maximally Tolerated Statin Therapy: the ODYSSEY COMBO I Study. Am. Heart J. 169 (6), 906–e13. 10.1016/j.ahj.2015.03.004 26027630

[B23] Kiyosue A. Honarpour N. Kurtz C. Xue A. Wasserman S. M. Hirayama A. (2016). A Phase 3 Study of Evolocumab (AMG 145) in Statin-Treated Japanese Patients at High Cardiovascular Risk. Am. J. Cardiol. 117 (1), 40–47. 10.1016/j.amjcard.2015.10.021 26547291

[B24] Kosmas C. E. Muñoz Estrella A. Sourlas A. Silverio D. Hilario E. Montan P. D. (2018). Inclisiran: A New Promising Agent in the Management of Hypercholesterolemia. Diseases 6 (3), 63. 10.3390/diseases6030063 PMC6163360 30011788

[B25] Kosmas C. E. Skavdis A. Sourlas A. Papakonstantinou E. J. Peña Genao E. Echavarria Uceta R. (2020). Safety and Tolerability of PCSK9 Inhibitors: Current Insights. Clin. Pharmacol. 12, 191–202. 10.2147/cpaa.S288831 33335431 PMC7737942

[B26] Landmesser U. Chapman M. J. Stock J. K. Amarenco P. Belch J. J. F. Borén J. (2018). 2017 Update of ESC/EAS Task Force on Practical Clinical Guidance for Proprotein Convertase Subtilisin/kexin Type 9 Inhibition in Patients with Atherosclerotic Cardiovascular Disease or in Familial Hypercholesterolaemia. Eur. Heart J. 39 (14), 1131–1143. 10.1093/eurheartj/ehx549 29045644

[B27] Lepor N. E. Kereiakes D. J. (2015). The PCSK9 Inhibitors: A Novel Therapeutic Target Enters Clinical Practice. Am. Health Drug Benefits 8 (9), 483–489. 26834934 PMC4719137

[B28] Li C. Lin L. Zhang W. Zhou L. Wang H. Luo X. (2015). Efficiency and Safety of Proprotein Convertase Subtilisin/kexin 9 Monoclonal Antibody on Hypercholesterolemia: a Meta-Analysis of 20 Randomized Controlled Trials. J. Am. Heart Assoc. 4 (6), e001937. 10.1161/JAHA.115.001937 26077586 PMC4599534

[B29] Lieb W. Enserro D. M. Larson M. G. Vasan R. S. (2018). Residual Cardiovascular Risk in Individuals on Lipid-Lowering Treatment: Quantifying Absolute and Relative Risk in the Community. Open Heart 5 (1), e000722. 10.1136/openhrt-2017-000722 29387429 PMC5786911

[B30] Lipinski M. J. Benedetto U. Escarcega R. O. Biondi-Zoccai G. Lhermusier T. Baker N. C. (2016). The Impact of Proprotein Convertase Subtilisin-Kexin Type 9 Serine Protease Inhibitors on Lipid Levels and Outcomes in Patients with Primary Hypercholesterolaemia: a Network Meta-Analysis. Eur. Heart J. 37 (6), 536–545. 10.1093/eurheartj/ehv563 26578202

[B31] Lloyd-Jones D. M. Lloyd-Jones D. M. Morris P. B. Ballantyne C. M. Birtcher K. K. Jr. DePalma S. M. (2016). 2016 ACC Expert Consensus Decision Pathway on the Role of Non-statin Therapies for LDL-Cholesterol Lowering in the Management of Atherosclerotic Cardiovascular Disease Risk: A Report of the American College of Cardiology Task Force on Clinical Expert Consensus Documents. J. Am. Coll. Cardiol. 68 (1), 92–125. 10.1016/j.jacc.2016.03.519 27046161

[B32] Macchi C. Ferri N. Sirtori C. R. Corsini A. Banach M. Ruscica M. (2021). Proprotein Convertase Subtilisin/Kexin Type 9: A View beyond the Canonical Cholesterol-Lowering Impact. Am. J. Pathol. 191 (8), 1385–1397. 10.1016/j.ajpath.2021.04.016 34019847

[B33] Mach F. Baigent C. Catapano A. L. Koskinas K. C. Casula M. Badimon L. (2020). 2019 ESC/EAS Guidelines for the Management of Dyslipidaemias: Lipid Modification to Reduce Cardiovascular Risk. Eur. Heart J. 41 (1), 111–188. 10.1093/eurheartj/ehz455 31504418

[B34] Mellwig K. P. Horstkotte D. van Buuren F. (2017). Lipoprotein (A) and Coronary Heart Disease - Is There an Efficient Secondary Prevention? Clin. Res. Cardiol. Suppl. 12 (Suppl. 1), 18–21. 10.1007/s11789-017-0088-x 28233270 PMC5352755

[B35] Myers K. D. Farboodi N. Mwamburi M. Howard W. Staszak D. Gidding S. (2019). Effect of Access to Prescribed PCSK9 Inhibitors on Cardiovascular Outcomes. Circ. Cardiovasc. Qual. Outcomes 12 (8), e005404. 10.1161/circoutcomes.118.005404 31331194 PMC7665275

[B36] Navarese E. P. Kolodziejczak M. Schulze V. Gurbel P. A. Tantry U. Lin Y. (2015). Effects of Proprotein Convertase Subtilisin/Kexin Type 9 Antibodies in Adults with Hypercholesterolemia: A Systematic Review and Meta-Analysis. Ann. Intern. Med. 163 (1), 40–51. 10.7326/m14-2957 25915661

[B37] Nelson C. P. Lai F. Y. Nath M. Ye S. Webb T. R. Schunkert H. (2019). Genetic Assessment of Potential Long-Term On-Target Side Effects of PCSK9 (Proprotein Convertase Subtilisin/Kexin Type 9) Inhibitors. Circ. Genom Precis Med. 12 (1), e002196. 10.1161/circgen.118.002196 30645167

[B38] Oesterle A. Laufs U. Liao J. K. (2017). Pleiotropic Effects of Statins on the Cardiovascular System. Circ. Res. 120 (1), 229–243. 10.1161/CIRCRESAHA.116.308537 28057795 PMC5467317

[B39] Page M. J. McKenzie J. E. Bossuyt P. M. Boutron I. Hoffmann T. C. Mulrow C. D. (2021). The PRISMA 2020 Statement: an Updated Guideline for Reporting Systematic Reviews. BMJ 372, n71. 10.1136/bmj.n71 33782057 PMC8005924

[B40] Peng W. Qiang F. Peng W. Qian Z. Ke Z. Yi L. (2016). Therapeutic Efficacy of PCSK9 Monoclonal Antibodies in Statin-Nonresponsive Patients with Hypercholesterolemia and Dyslipidemia: A Systematic Review and Meta-Analysis. Int. J. Cardiol. 222, 119–129. 10.1016/j.ijcard.2016.07.239 27494723

[B41] Raal F. Scott R. Somaratne R. Bridges I. Li G. Wasserman S. M. (2012). Low-density Lipoprotein Cholesterol-Lowering Effects of AMG 145, a Monoclonal Antibody to Proprotein Convertase Subtilisin/kexin Type 9 Serine Protease in Patients with Heterozygous Familial Hypercholesterolemia: the Reduction of LDL-C with PCSK9 Inhibition in Heterozygous Familial Hypercholesterolemia Disorder (RUTHERFORD) Randomized Trial. Circulation 126 (20), 2408–2417. 10.1161/circulationaha.112.144055 23129602

[B42] Raal F. J. Stein E. A. Dufour R. Turner T. Civeira F. Burgess L. (2015). PCSK9 Inhibition with Evolocumab (AMG 145) in Heterozygous Familial Hypercholesterolaemia (RUTHERFORD-2): a Randomised, Double-Blind, Placebo-Controlled Trial. Lancet 385 (9965), 331–340. 10.1016/s0140-6736(14)61399-4 25282519

[B43] Raal F. J. Honarpour N. Blom D. J. Hovingh G. K. Xu F. Scott R. (2015). Inhibition of PCSK9 with Evolocumab in Homozygous Familial Hypercholesterolaemia (TPMIDESLA Part B): A Randomised, Double-Blind, Placebo-Controlled Trial. Lancet 385 (9965), 341–350. 10.1016/S0140-6736(14)61374-X 25282520

[B44] Ray K. K. Landmesser U. Leiter L. A. Kallend D. Dufour R. Karakas M. (2017). Inclisiran in Patients at High Cardiovascular Risk with Elevated LDL Cholesterol. N. Engl. J. Med. 376 (15), 1430–1440. 10.1056/NEJMoa1615758 28306389

[B45] Ray K. K. Wright R. S. Kallend D. Koenig W. Leiter L. A. Raal F. J. (2020). Two Phase 3 Trials of Inclisiran in Patients with Elevated LDL Cholesterol. N. Engl. J. Med. 382 (16), 1507–1519. 10.1056/NEJMoa1912387 32187462

[B46] Ridker P. M. Danielson E. Fonseca F. A. Genest J. Gotto A. M. Jr. Kastelein J. J. (2008). Rosuvastatin to Prevent Vascular Events in Men and Women with Elevated C-Reactive Protein. N. Engl. J. Med. 359 (21), 2195–2207. 10.1056/NEJMoa0807646 18997196

[B47] Robinson J. G. Nedergaard B. S. Rogers W. J. Fialkow J. Neutel J. M. Ramstad D. (2014). Effect of Evolocumab or Ezetimibe Added to Moderate- or High-Intensity Statin Therapy on LDL-C Lowering in Patients with Hypercholesterolemia: the LAPLACE-2 Randomized Clinical Trial. Jama 311 (18), 1870–1882. 10.1001/jama.2014.4030 24825642

[B48] Robinson J. G. Farnier M. Krempf M. Bergeron J. Luc G. Averna M. (2015). Efficacy and Safety of Alirocumab in Reducing Lipids and Cardiovascular Events. N. Engl. J. Med. 372 (16), 1489–1499. 10.1056/NEJMoa1501031 25773378

[B49] Robinson J. G. Jayanna M. B. Bairey Merz C. N. Stone N. J. (2020). Clinical Implications of the Log Linear Association between LDL-C Lowering and Cardiovascular Risk Reduction: Greatest Benefits when LDL-C >100 Mg/dl. PLOS ONE 15 (10), e0240166. 10.1371/journal.pone.0240166 33119602 PMC7595281

[B50] Roth E. M. Moriarty P. M. Bergeron J. Langslet G. Manvelian G. Zhao J. (2016). A Phase III Randomized Trial Evaluating Alirocumab 300 Mg Every 4 Weeks as Monotherapy or Add-On to Statin: ODYSSEY CHOICE I. Atherosclerosis 254, 254–262. 10.1016/j.atherosclerosis.2016.08.043 27639753

[B51] Sabatine M. S. Giugliano R. P. Keech A. C. Honarpour N. Wiviott S. D. Murphy S. A. (2017). Evolocumab and Clinical Outcomes in Patients with Cardiovascular Disease. N. Engl. J. Med. 376 (376), 1713–1722. 10.1056/NEJMoa1615664 28304224

[B52] Sabatine M. S. (2019). PCSK9 Inhibitors: Clinical Evidence and Implementation. Nat. Rev. Cardiol. 16 (3), 155–165. 10.1038/s41569-018-0107-8 30420622

[B53] Sacks F. M. (2006). The Apolipoprotein story. Atheroscler. Suppl. 7 (4), 23–27. 10.1016/j.atherosclerosissup.2006.05.004 16822722

[B54] Salanti G. Higgins J. P. Ades A. E. Ioannidis J. P. (2008). Evaluation of Networks of Randomized Trials. Stat. Methods Med. Res. 17 (3), 279–301. 10.1177/0962280207080643 17925316

[B55] Salanti G. Ades A. E. Ioannidis J. P. (2011). Graphical Methods and Numerical Summaries for Presenting Results from Multiple-Treatment Meta-Analysis: an Overview and Tutorial. J. Clin. Epidemiol. 64 (2), 163–171. 10.1016/j.jclinepi.2010.03.016 20688472

[B56] Schmidt A. F. Pearce L. S. Wilkins J. T. Overington J. P. Hingorani A. D. Casas J. P. (2017). PCSK9 Monoclonal Antibodies for the Primary and Secondary Prevention of Cardiovascular Disease. Cochrane Database Syst. Rev. 4 (4), Cd011748. 10.1002/14651858.CD011748.pub2 28453187 PMC6478267

[B57] Abdullah S. M. Defina L. F. Leonard D. Barlow C. E. Radford N. B. Willis B. L. (2018). Long-Term Association of Low-Density Lipoprotein Cholesterol with Cardiovascular Mortality in Individuals at Low 10-Year Risk of Atherosclerotic Cardiovascular Disease. Circulation, 138(21), 2315–2325. 10.1161/CIRCULATIONAHA.118.034273 30571575

[B58] Stamler J. Neaton J. D. (2008). The Multiple Risk Factor Intervention Trial (MRFIT)--importance Then and Now. JAMA 300 (11), 1343–1345. 10.1001/jama.300.11.1343 18799447

[B59] Strilchuk L. Fogacci F. Cicero A. F. (2019). Safety and Tolerability of Injectable Lipid-Lowering Drugs: an Update of Clinical Data. Expert Opin. Drug Saf. 18 (7), 611–621. 10.1080/14740338.2019.1620730 31100030

[B60] Teramoto T. Kobayashi M. Tasaki H. Yagyu H. Higashikata T. Takagi Y. (2016). Efficacy and Safety of Alirocumab in Japanese Patients with Heterozygous Familial Hypercholesterolemia or at High Cardiovascular Risk with Hypercholesterolemia Not Adequately Controlled with Statins - ODYSSEY JAPAN Randomized Controlled Trial. Circ. J. 80 (9), 1980–1987. 10.1253/circj.CJ-16-0387 27452202

[B61] Toth P. P. Worthy G. Gandra S. R. Sattar N. Bray S. Cheng L. I. (2017). Systematic Review and Network Meta-Analysis on the Efficacy of Evolocumab and Other Therapies for the Management of Lipid Levels in Hyperlipidemia. J. Am. Heart Assoc. 6 (10), e005367. 10.1161/jaha.116.005367 28971955 PMC5721820

[B62] Tu Y.-K. (2015). Using Generalized Linear Mixed Models to Evaluate Inconsistency within a Network Meta-Analysis. Value in Health 18 (8), 1120–1125. 10.1016/j.jval.2015.10.002 26686799

[B63] Yu-Kang T. (2016). Node-Splitting Generalized Linear Mixed Models for Evaluation of Inconsistency in Network Meta-Analysis. Value Health 19 (8), 957–963. 10.1016/j.jval.2016.07.005 27987646

[B64] Zhang X. L. Zhu Q. Q. Zhu L. Chen J. Z. Chen Q. H. Li G. N. (2015). Safety and Efficacy of Anti-PCSK9 Antibodies: a Meta-Analysis of 25 Randomized, Controlled Trials. BMC Med. 13 (1), 123. 10.1186/s12916-015-0358-8 26099511 PMC4477483

