# Supplementary material for: Efficacy and Safety of Proprotein Convertase Subtilisin/Kexin Type 9 Inhibitors as Adjuvant Treatments for Patients with Hypercholesterolemia Treated with Statin: A Systematic Review and Network Meta-analysis
Source: Front Pharmacol. 2022 Apr 4;13:832614. doi: 10.3389/fphar.2022.832614 (PMC9014015; doi:10.3389/fphar.2022.832614)
Supplement: Supplementary file 1 [file DataSheet1.docx]

Supplementary Materials

**Efficacy and Safety of Proprotein Convertase Subtilisin/Kexin Type 9 Inhibitors as Adjuvant Treatments for Patients with Hypercholesterolemia Treated with Statin: a Systematic Review and Network Meta-analysis**

Yi-Ting Huang, MS^a^; Li-Ting Ho, MD^a,b^; Hsin-Yin Hsu MD, MS^a,c,d^; Yu-Kang Tu, DDS, PhD^a,b,*^; Kuo-Liong Chien, MD, PhD^a,b,*^

^a^ Institute of Epidemiology and Preventive Medicine, College of Public Health, National Taiwan University, Taipei, Taiwan;

^b^ Department of Internal Medicine, National Taiwan University Hospital and College of Medicine, Taipei, Taiwan

^c^ Department of Family Medicine, Taipei MacKay Memorial Hospital, Taipei, Taiwan

^d^ Department of Medicine, MacKay Medical College, New Taipei City, Taiwan

***Corresponding authors:**

Kuo-Liong Chien^1^ and Yu-Kang Tu^2^ contributed equally to this work.

Institute of Epidemiology and Preventive Medicine, College of Public Health, National Taiwan University

No. 17, Hsu Chow Road, Taipei, Taiwan, 100

^1^Telephone: +886-2-3366-8017; Fax: +886-2-2351-1955

E-mail: klchien@ntu.edu.tw

^2^Telephone: +886-2-3366-8039; Fax: +886-2-2351-1955

E-mail: yukangtu@ntu.edu.tw

**Table of contents**

Supplementary Appendix 1. Study protocol and search strategies

Supplementary Figure 1. Risk of bias summary for included studies

Supplementary Figure 2. Risk of bias graph of included studies

Supplementary Figure 3. Network geometry of apolipoprotein B and lipoprotein(a)

Supplementary Figure 4. Network geometry of adverse events

Supplementary Figure 5. Results of network meta-analysis for percentage change in lipids between PCSK9 inhibitors/ezetimibe and placebo.

Supplementary Figure 6. Results of network meta-analysis for odds ratio of adverse event between PCSK9 inhibitors/ezetimibe and placebo

Supplementary Figure 7. Direct and indirect evidence for estimating the percentage change in low-density lipoprotein cholesterol

Supplementary Figure 8. Direct and indirect evidence for estimating the percentage change in apolipoprotein B

Supplementary Figure 9. Direct and indirect evidence for estimating the percentage change in lipoprotein(a)

Supplementary Figure 10. Direct and indirect evidence for estimating the percentage change in low-density lipoprotein cholesterol

Supplementary Figure 11. Direct and indirect evidence for estimating the percentage change in apolipoprotein B

Supplementary Figure 12. Direct and indirect evidence for estimating the percentage change in lipoprotein(a)

Supplementary Figure 13. Direct and indirect evidence for estimating the odds ratio of adverse event

Supplementary Figure 14. Direct and indirect evidence for estimating the odds ratio of severe adverse event

Supplementary Figure 15. Direct and indirect evidence for estimating the odds ratio of adverse event (treatment with different drug doses)

Supplementary Figure 16. Direct and indirect evidence for estimating the odds ratio of nasopharyngitis event (treatment with different drug doses)

Supplementary Figure 17. Direct and indirect evidence for estimating the odds ratio of injection-site reaction (treatment with different drug doses)

Supplementary Figure 18. Direct and indirect evidence for estimating the odds ratio of severe adverse event (treatment with different drug doses)

Supplementary Figure 19. The assessment of publication bias for percentage change in low-density lipoprotein cholesterol

Supplementary Figure 20. The assessment of publication bias for percentage change in apolipoprotein B

Supplementary Figure 21. The assessment of publication bias for percentage change in lipoprotein(a)

Supplementary Figure 22. The assessment of publication bias for odds ratio of adverse event

Supplementary Figure 23. The assessment of publication bias for odds ratio of nasopharyngitis event

Supplementary Figure 24. The assessment of publication bias for odds ratio of injection-site reaction

Supplementary Figure 25. The assessment of publication bias for odds ratio of serious adverse event

Supplementary Figure 26. Sensitivity analysis of lipid change between PCSK9 inhibitors/ezetimibe and placebo using an alternative meta-analysis model, fixed-effect model

Supplementary Figure 27. Sensitivity analysis of adverse event between PCSK9 inhibitors/ezetimibe and placebo using an alternative meta-analysis model, fixed-effect model

Supplementary Table 1. Result of inconsistency testing

Supplementary Table 2-1. The checklist of preferring reporting items for systematic reviews and meta-analyses

Supplementary Table 2-2. PRISMA 2020 for abstracts checklist

**Supplementary Appendix 1. Study protocol and search strategies**

**Objective**

To evaluate all classes and dosages of Proprotein Convertase Subtilisin/Kexin Type 9 (PCSK9) inhibitors which serve as an add-on statin therapy for hypercholesterolemia patients.

**PICOS criteria for inclusion of studies**

**Participants**

Patients older than 18 years old with low-density lipoprotein (LDL) cholesterol greater than 70 mg/dl, hypercholesterolemia, hyperlipidemia, mixed dyslipidemia, or a high cardiovascular risk.

**Interventions and Comparators**

Interventions and comparators will be different classes and dosages of PCSK9 inhibitors, and placebo or ezetimibe, which is used as an adjuvant therapy of statin.

For a dose-ranging study with different dosages of PCSK9 inhibitor, we will include those dosages approved by FDA; when the PCSK9 inhibitor is not yet approved by FDA, we will include the dosages with the greatest reduction in LDL cholesterol into the network meta-analysis.

**Outcomes**

Efficacy outcomes: LDL cholesterol, apolipoprotein B or lipoprotein(a)

Safety outcomes: adverse event, nasopharyngitis, injection-site reaction, serious adverse event

**Study design**

The study design will be randomized controlled phase 3 trials with parallel design that randomly allocate patients treated with statin to at least two of the following treatments: one of different classes and dosages of PCSK9 inhibitors, placebo, or ezetimibe. The eligible study should not be a duplicate study.

**Data extraction and quality assessment**

Eligible articles fulfilling the inclusion criteria will be included in this study irrespective of their language. Two investigators (Yi-Ting Huang and Li-Ting Ho) will extract the following information: trial name, published year, study population, duration of follow-up, characteristic of patient (age, sex, type 2 diabetes, baseline LDL cholesterol, baseline apolipoprotein B, baseline lipoprotein(a)), percentage change from baseline to follow-up time and its standard deviation (or standard error of the mean) in LDL cholesterol, apolipoprotein B, and lipoprotein(a), and the number of patients who suffered adverse event, nasopharyngitis, injection-site reaction, serious adverse event, and cardiovascular outcomes. If data required for our review is incomplete or lack sufficient details, we will contact the original authors to request further information by email.

Eligible articles will be independently evaluated for the quality assessment by two investigators (Yi-Ting Huang and Li-Ting Ho). We use The Cochrane Collaboration’s tool for assessing risk of bias to assess selection bias, performance bias, detection bias, attrition bias, reporting bias, and other bias in the article. If there is any concern or difference between results of assessments, a senior investigator (Yu-Kang Tu) would be consulted. We used risk of bias graph to illustrate the results of potential risk of bias regarding percentage of unclear, low risk or high risk.

**Data Synthesis and Analysis**

The weighted mean differences in LDL cholesterol, apolipoprotein B, and lipoprotein(a) changes with the corresponding 95% confidence interval are to be estimated for the efficacy of different PCSK9 inhibitors relative to placebo or ezetimibe. Odds ratio with corresponding 95% confidence interval is estimated for the differences in the incidences of adverse events, nasopharyngitis, injection-site reaction, neurological events, and serious adverse events.

We will conduct network meta-analyses, proposed by Lu and Ades, to combine direct evidence and indirect evidence to compare different classes and dosages of PCSK9 inhibitors.

For ranking the treatments, we will calculate the probabilities of being in different positions for each treatment by undertaking 1000 simulations. The simulations will be set up by using the estimated regression coefficients and their variance-covariance matrix to generate hypothetical datasets. Treatment effects will estimate in each simulated dataset are then use to calculate the ranking for each treatment. Moreover, consistency assumption within our network meta-analyses is evaluated by the design-by-treatment interaction model and node-splitting model, and the Wald test for testing hypotheses about inconsistency. Design-by-treatment interaction model is to examine the consistency in the effects of treatments in trials of different designs, where the design represents the set of treatments compared within a trial. Node-splitting model is to examine the consistency in the effects of treatments between direct and indirect comparisons. Furthermore, we will use funnel plot and Egger’s test to examine the publication bias for each pairwise comparison. All statistical analyses will be performed with R statistical software.

**Search strategies**

We searched the following electronic databases up to April 20, 2021.

1. **PubMed**

*(hypercholesterolemia OR hypercholesterolaemia OR cholesteremia OR cholesterinemia OR cholesterolemia OR hypercholesteremia OR hypercholesterinaemia OR hypercholesterinemia) AND (“hydroxymethylglutaryl coenzyme A reductase inhibitor” OR “HMG*CoA reductase inhibitor” OR *Statin) AND (“proprotein convertase subtilisin*kexin type 9 inhibitor” OR “PCSK9 inhibitor” OR alirocumab OR REGN727 OR SAR236553 OR Praluent OR evolocumab OR “AMG*145” OR Repatha OR bococizumab OR RN316 OR “PF-04950615” OR frovocimab OR LY3015014 OR inclisiran OR “ALN*PCSsc” OR RG7652 OR MPSK3169A OR Ebronucimab OR AK102 OR JS002 OR Lerodalcibep OR IBI306 OR CIVI007)*

*)*

1. **Embase**

(hypercholesterolemia OR hypercholesterolaemia OR cholesteremia OR cholesterinemia OR cholesterolemia OR hypercholesteremia OR hypercholesterinaemia OR hypercholesterinemia) AND (‘hydroxymethylglutaryl coenzyme A reductase inhibitor’ OR ‘HMG*CoA reductase inhibitor’) AND (‘proprotein convertase subtilisin*kexin type 9 inhibitor’ OR ‘PCSK9 inhibitor’ OR alirocumab OR REGN727 OR SAR236553 OR Praluent OR evolocumab OR ‘AMG*145’ OR Repatha OR bococizumab OR RN316 OR ‘PF-04950615’ OR frovocimab OR LY3015014 OR inclisiran OR ‘ALN*PCSsc’ *OR RG7652 OR MPSK3169A OR Ebronucimab OR AK102 OR JS002 OR Lerodalcibep OR IBI306 OR CIVI007*)

1. **Cochrane Library**

*(hypercholesterolemia OR hypercholesterolaemia OR cholesteremia OR cholesterinemia OR cholesterolemia OR hypercholesteremia OR hypercholesterinaemia OR hypercholesterinemia) AND (“hydroxymethylglutaryl coenzyme A reductase inhibitor” OR “HMG*CoA reductase inhibitor” OR *Statin) AND (“proprotein convertase subtilisin*kexin type 9 inhibitor” OR “PCSK9 inhibitor” OR alirocumab OR REGN727 OR SAR236553 OR Praluent OR evolocumab OR AMG*145 OR Repatha OR bococizumab OR RN316 OR PF-04950615 OR frovocimab OR LY3015014 OR inclisiran OR ALN*PCSsc OR RG7652 OR MPSK3169A OR Ebronucimab OR AK102 OR JS002 OR Lerodalcibep OR IBI306 OR CIVI007)*

1. **Web of Science**

*(hypercholesterolemia OR hypercholesterolaemia OR cholesteremia OR cholesterinemia OR cholesterolemia OR hypercholesteremia OR hypercholesterinaemia OR hypercholesterinemia) AND (“hydroxymethylglutaryl coenzyme A reductase inhibitor” OR “HMG*CoA reductase inhibitor” OR *Statin) AND (“proprotein convertase subtilisin*kexin type 9 inhibitor” OR “PCSK9 inhibitor” OR alirocumab OR REGN727 OR SAR236553 OR Praluent OR evolocumab OR “AMG*145” OR Repatha OR bococizumab OR RN316 OR “PF-04950615” OR frovocimab OR LY3015014 OR inclisiran OR “ALN*PCSsc” OR RG7652 OR MPSK3169A OR Ebronucimab OR AK102 OR JS002 OR Lerodalcibep OR IBI306 OR CIVI007)*

1. **ClinicalTrials.gov**

Recruitment: All studies

Study type: Interventional studies (Clinical trials)

Study Results: All studies

Conditions: Hypercholesterolemia

Interventions: *Statin AND (PCSK9 inhibitor OR alirocumab OR evolocumab OR bococizumab OR LY3015014 OR inclisiran OR RG7652 OR Ebronucimab OR AK102 OR JS002 OR Lerodalcibep OR IBI306 OR CIVI007)*

Study phase: phase 3

Eligibility criteria: adult (>=18)

**Supplementary Figure 1. Risk of bias summary for included studies**

Results of quality assessment for each of included studies according to the items of risk of bias. Color labels: Green for low risk of bias; Yellow for unclear; Red for high risk of bias.


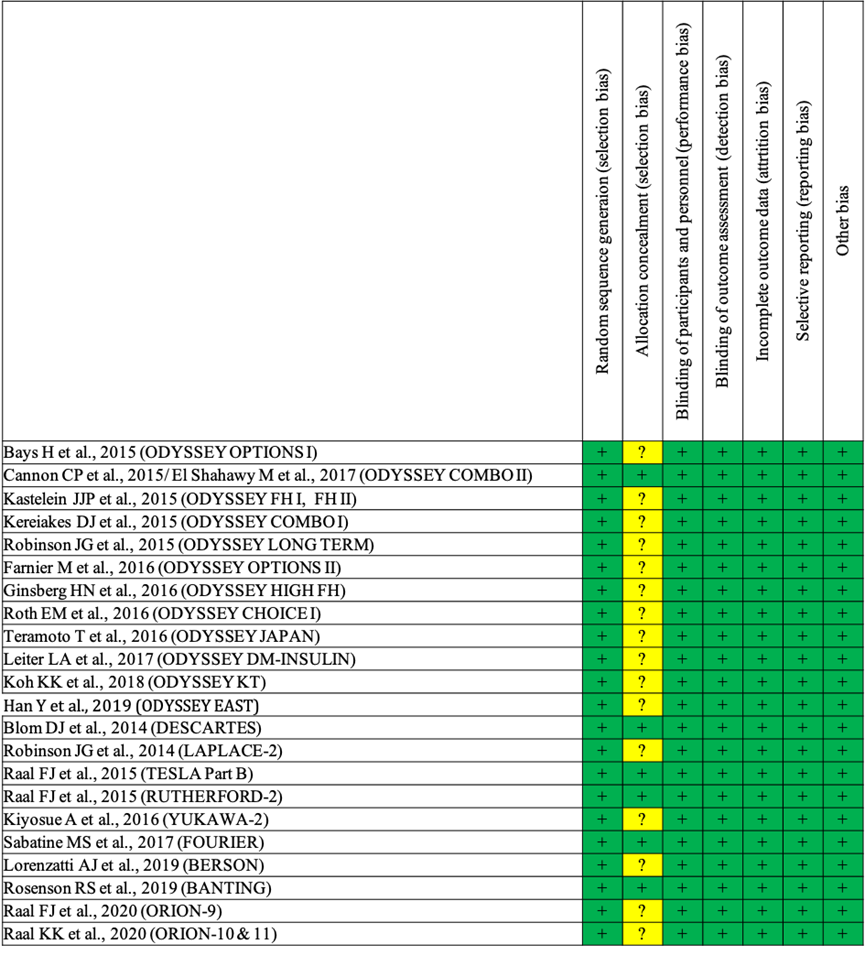


**Supplementary Figure 2. Risk of bias graph of included studies**

Percentages of included studies with different levels of the risk of bias.


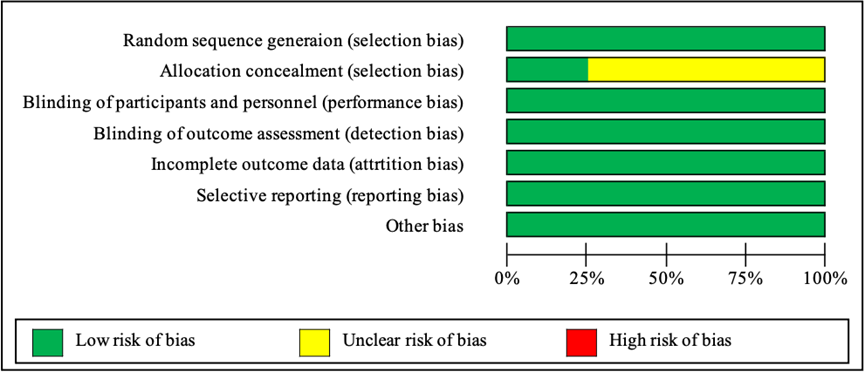


**Supplementary Figure 3. Network geometry of apolipoprotein B and lipoprotein(a).**

The sizes of treatment nodes reflect the number of patients randomly assign to each treatment. The thicknesses of edges represent the number of studies underlying each comparison.

| Treatment | Treatment with different drug doses |
| --- | --- |
| **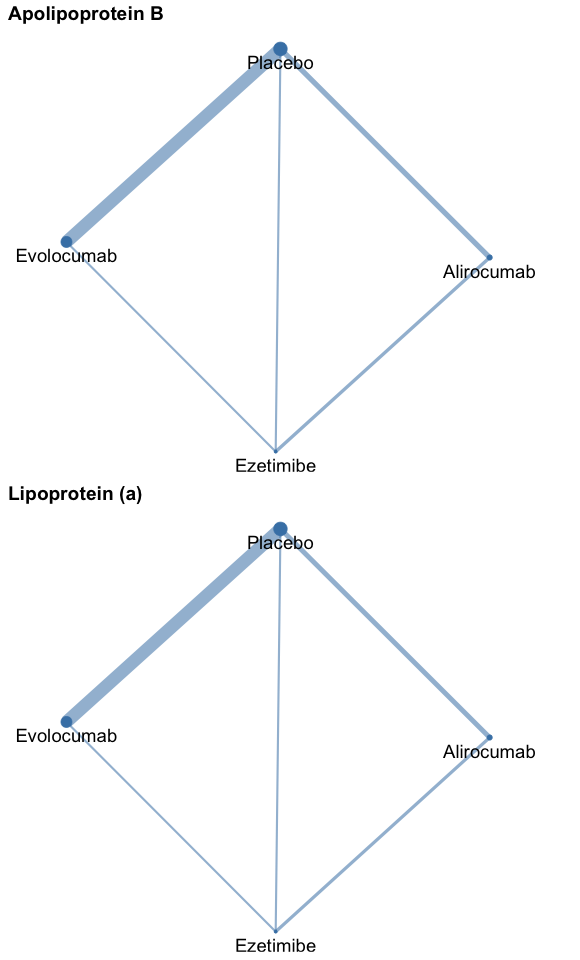** | **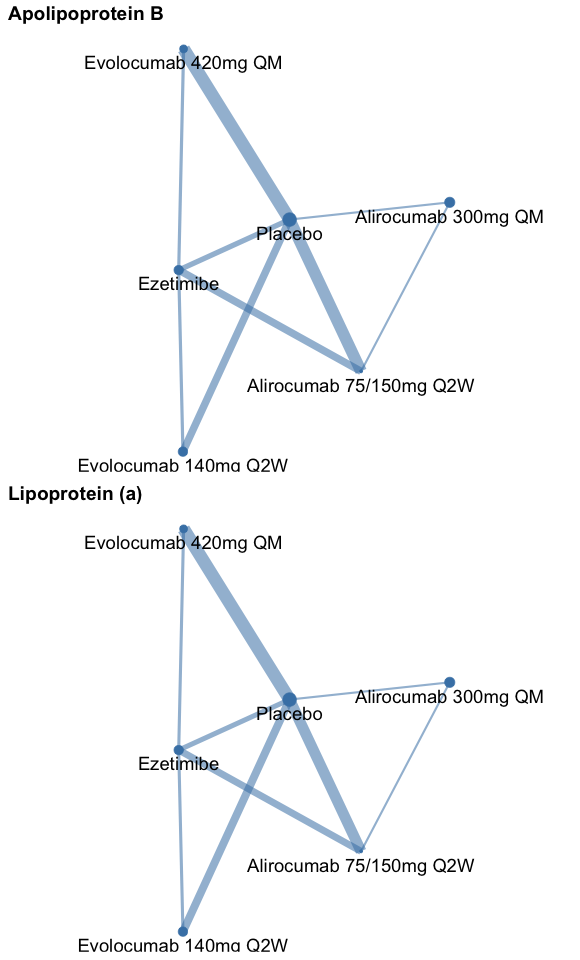** |

**Supplementary Figure 4. Network geometry of adverse events**

The sizes of treatment nodes reflect the number of patients randomly assign to each treatment. The thicknesses of edges represent the number of studies underlying each comparison.

| Treatment | Treatment with different drug doses |
| --- | --- |
| **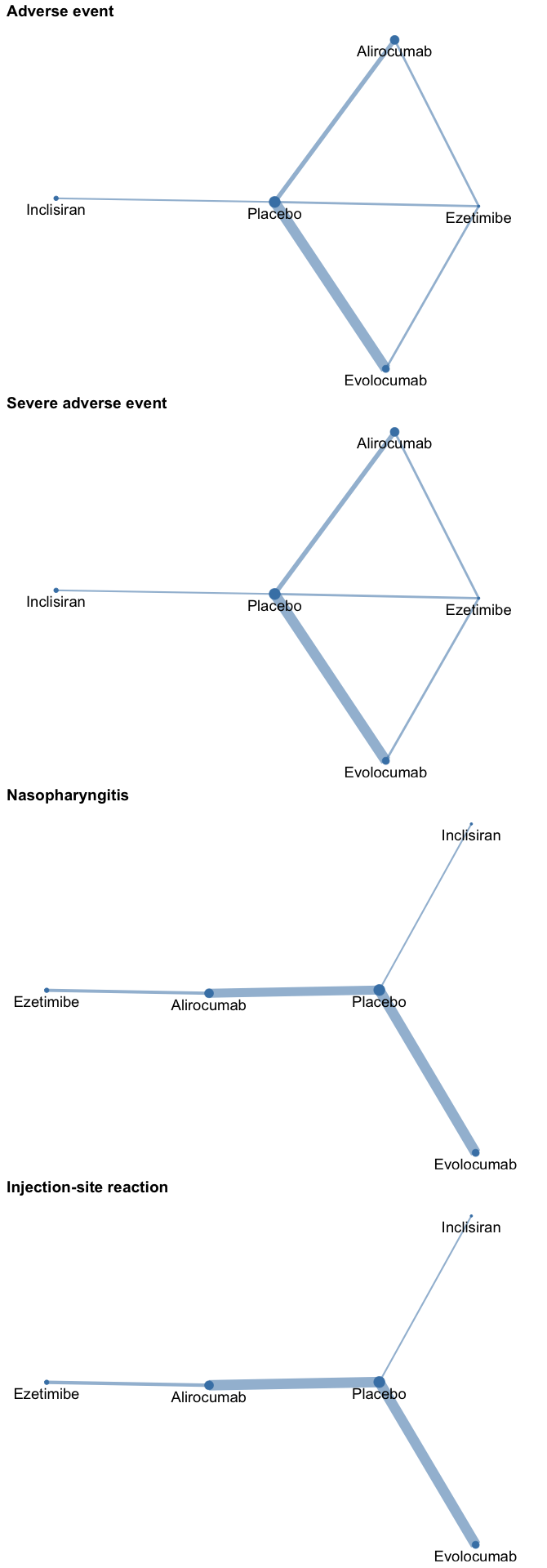** | **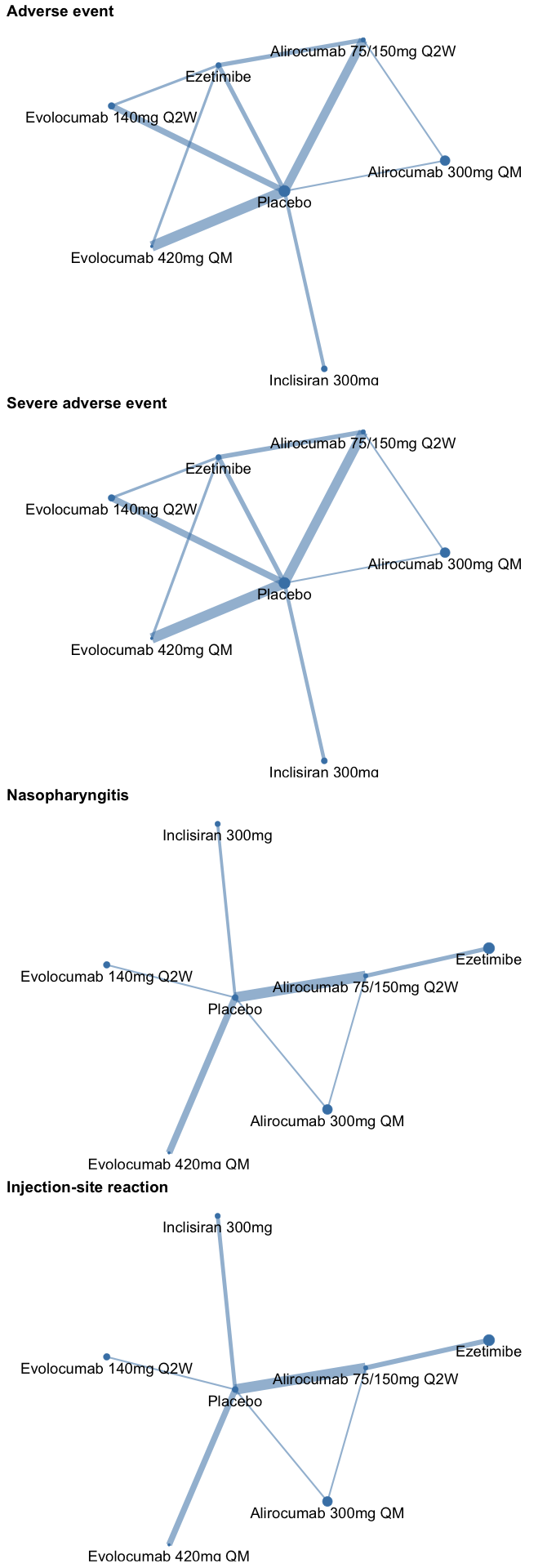** |

**Supplementary Figure 5. Results of network meta-analysis for percentage change in lipids between PCSK9 inhibitors/ezetimibe and placebo.**


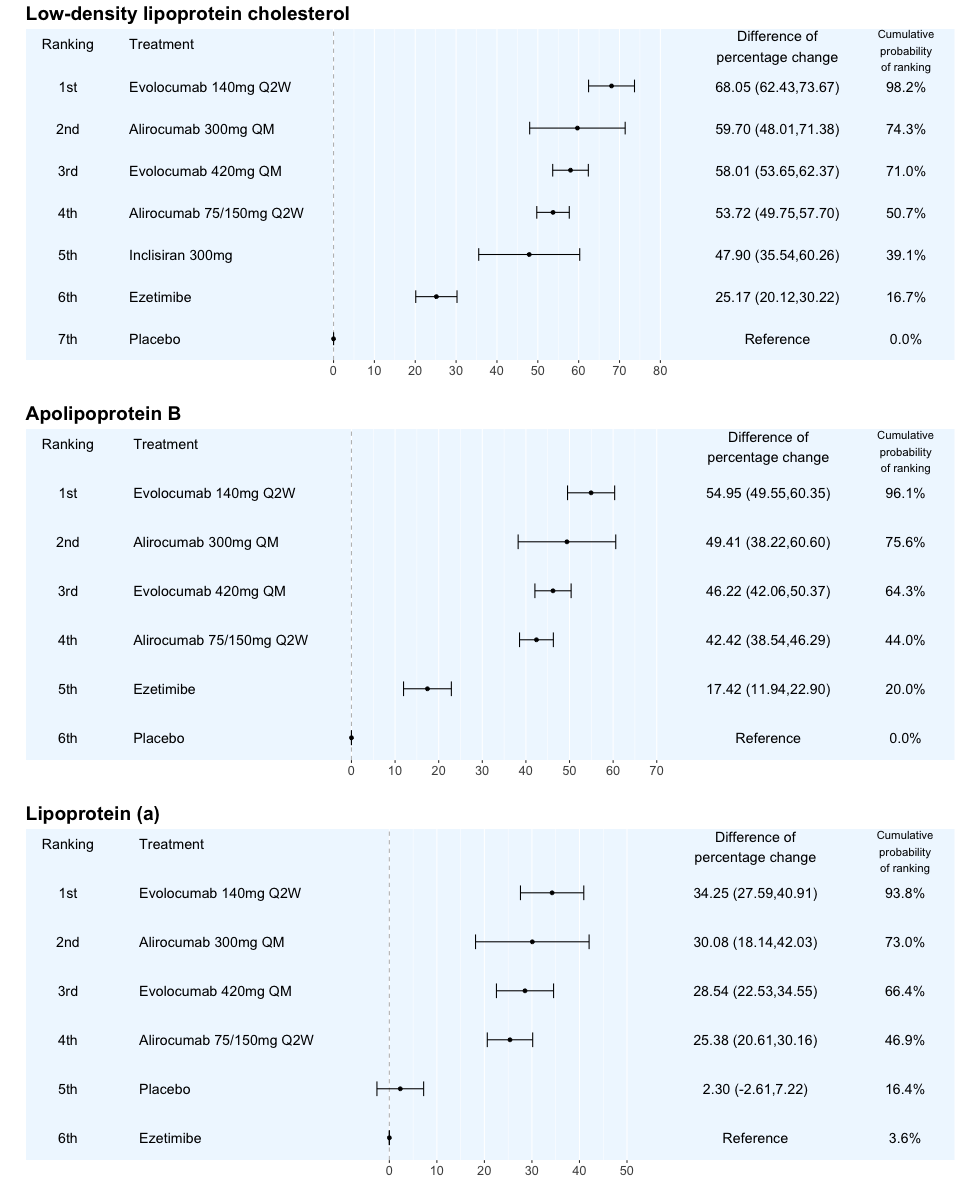


**Supplementary Figure 6. Results of network meta-analysis for odds ratio of adverse event between PCSK9 inhibitors/ezetimibe and placebo**


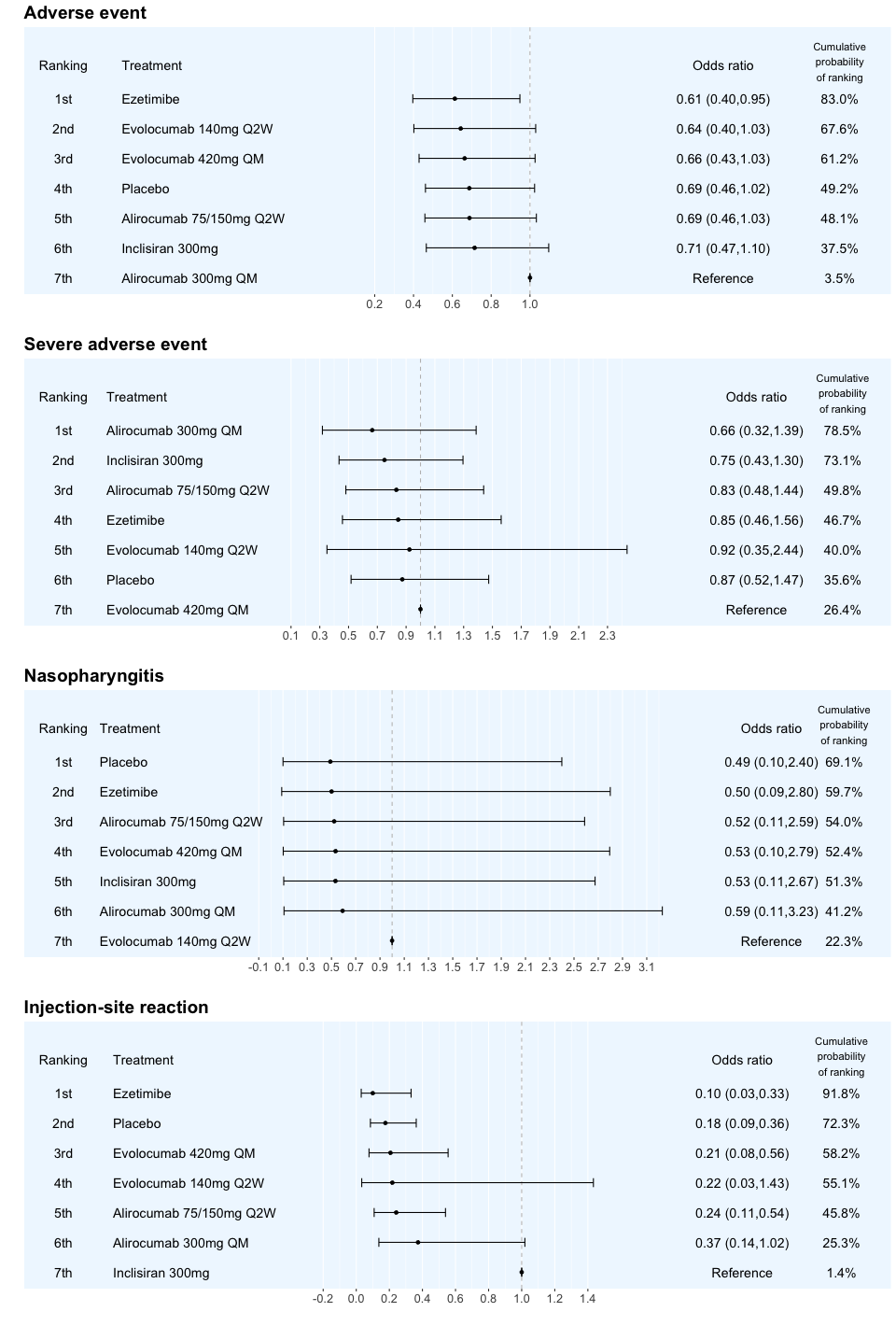


**Supplementary Figure 7. Direct and indirect evidence for estimating the percentage change in low-density lipoprotein cholesterol**

**
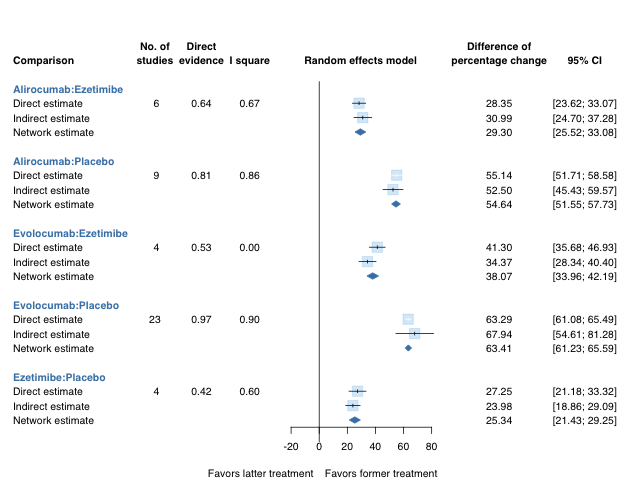
**

**Supplementary Figure 8. Direct and indirect evidence for estimating the percentage change in apolipoprotein B**

**
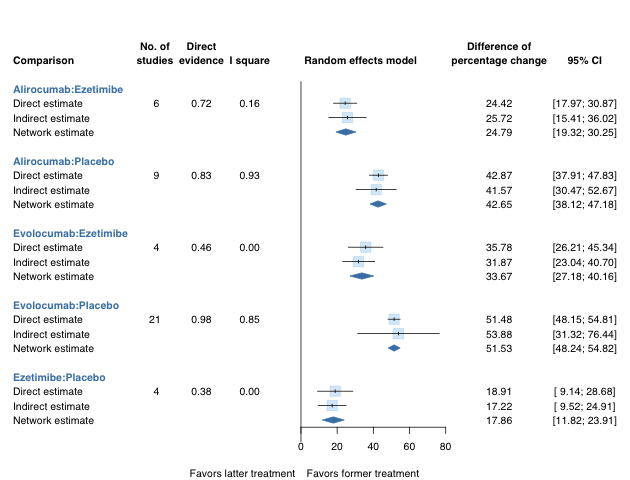
**

**Supplementary Figure 9. Direct and indirect evidence for estimating the percentage change in lipoprotein(a)**

**
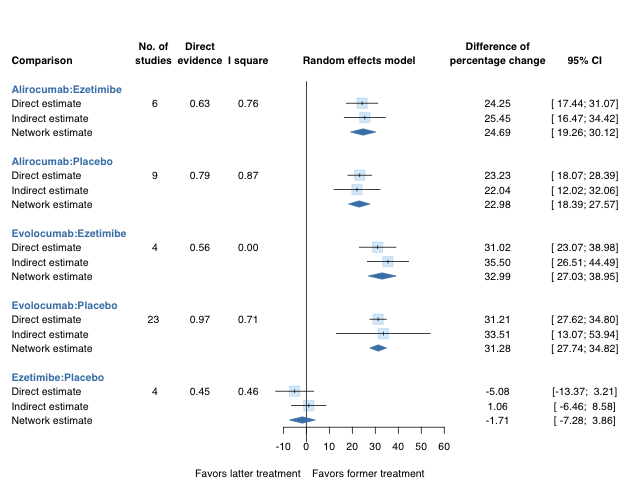
**

**Supplementary Figure 10. Direct and indirect evidence for estimating the percentage change in low-density lipoprotein cholesterol**

**
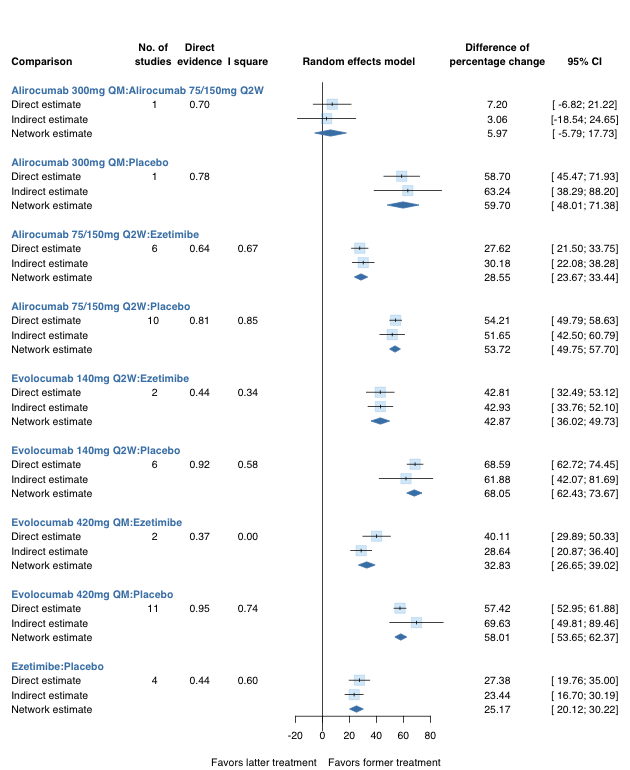
**

**Supplementary Figure 11. Direct and indirect evidence for estimating the percentage change in apolipoprotein B**

**
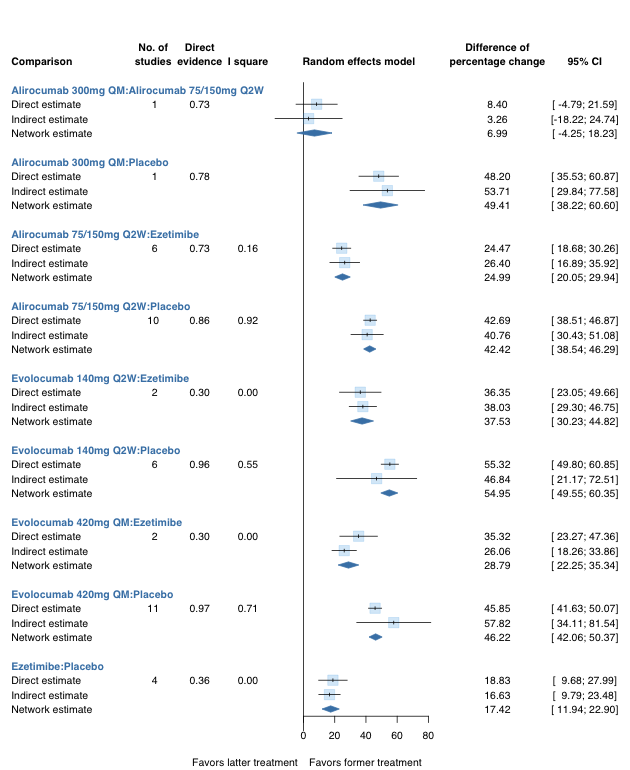
**

**Supplementary Figure 12. Direct and indirect evidence for estimating the percentage change in lipoprotein(a)**

**
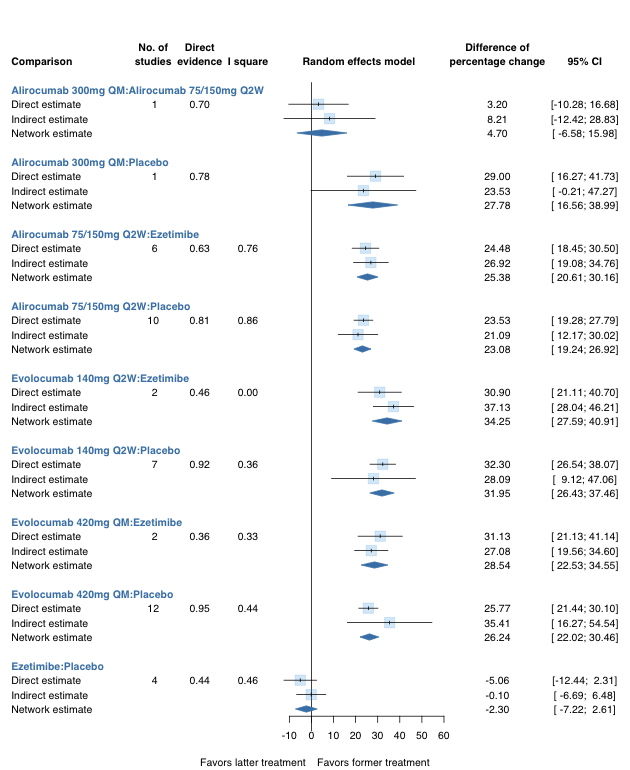
**

**Supplementary Figure 13. Direct and indirect evidence for estimating the odds ratio of adverse event**

**
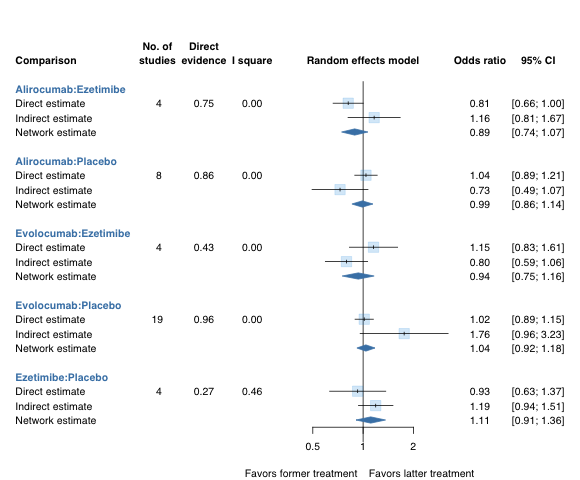
**

**Supplementary Figure 14. Direct and indirect evidence for estimating the odds ratio of severe adverse event**

**
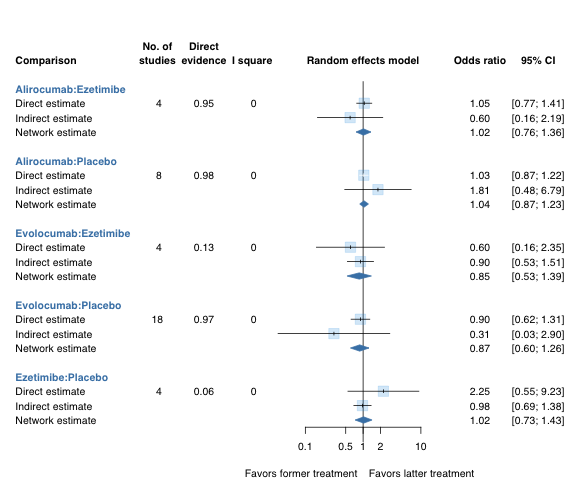
**

**Supplementary Figure 15. Direct and indirect evidence for estimating the odds ratio of adverse event**

**
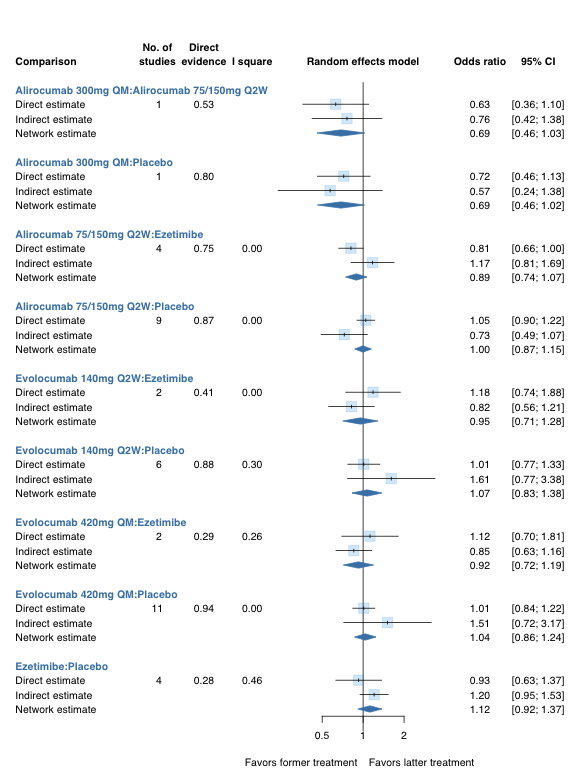
**

**Supplementary Figure 16. Direct and indirect evidence for estimating the odds ratio of nasopharyngitis event**

**
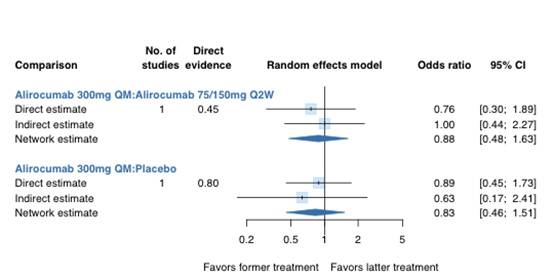
**

**Supplementary Figure 17. Direct and indirect evidence for estimating the odds ratio of injection-site reaction**

**
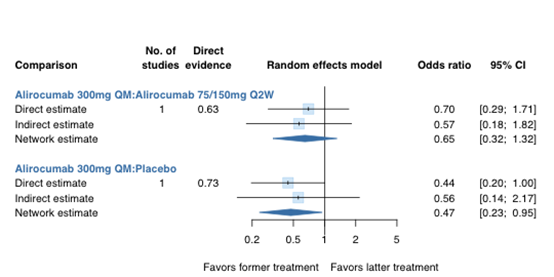
**

**Supplementary Figure 18. Direct and indirect evidence for estimating the odds ratio of severe adverse event**

**
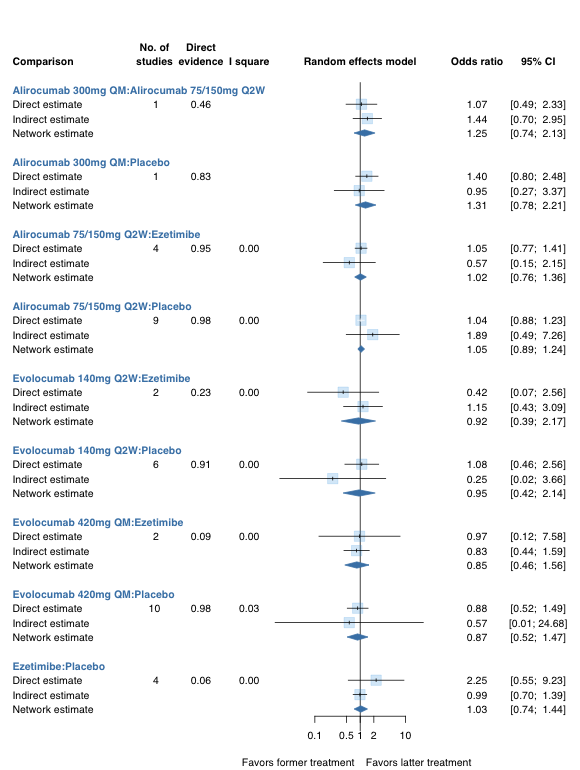
**

**Supplementary Figure 19. The assessment of publication bias for percentage change in low-density lipoprotein cholesterol**

In funnel plot, each point represents a treatment contrast reported by a study. Asymmetry in the distribution of data points in a funnel plot may suggest publication bias. The dashed lines in funnel plot represent the 95% confidence interval given the standard error of the effect size, and 95% of the included studies should be within the triangular region. For network meta-analysis, reported effect sizes have been centered, so the solid vertical line is located at zero.


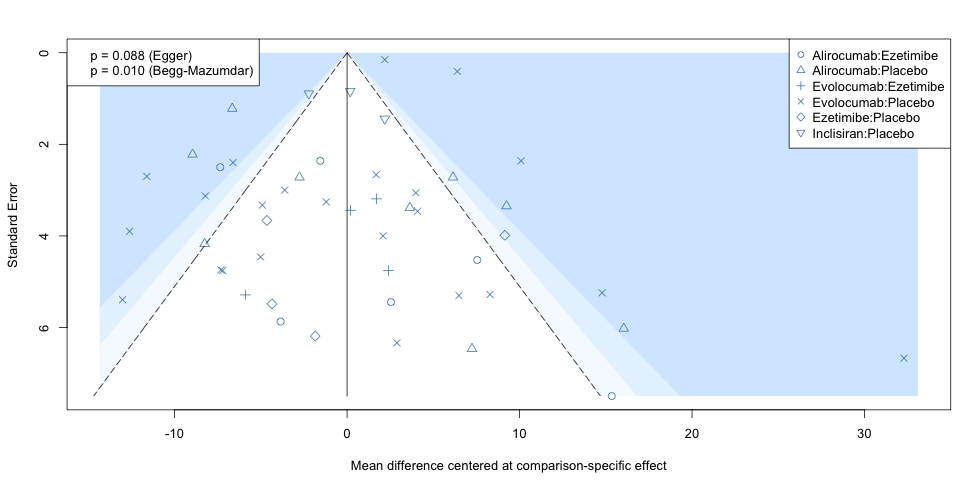


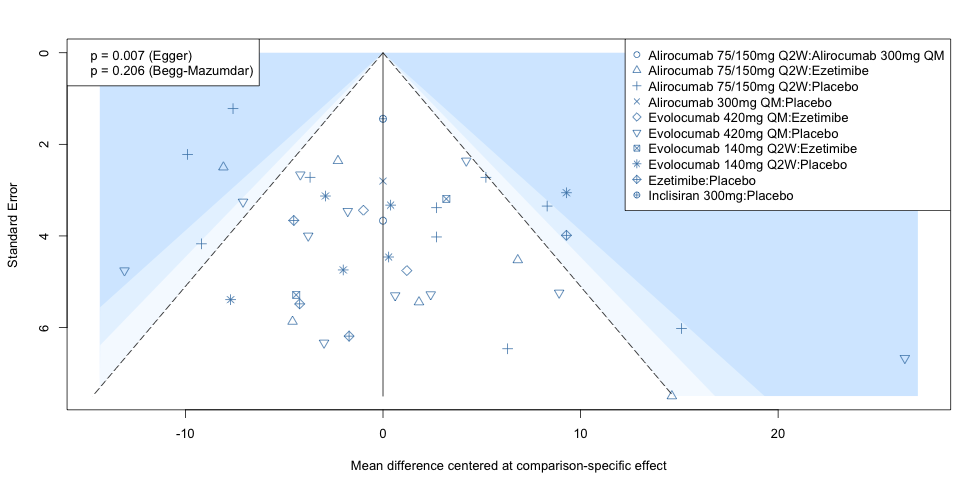


**Supplementary Figure 20. The assessment of publication bias for percentage change in apolipoprotein B**

In funnel plot, each point represents a treatment contrast reported by a study. Asymmetry in the distribution of data points in a funnel plot may suggest publication bias. The dashed lines in funnel plot represent the 95% confidence interval given the standard error of the effect size, and 95% of the included studies should be within the triangular region. For network meta-analysis, reported effect sizes have been centered, so the solid vertical line is located at zero.


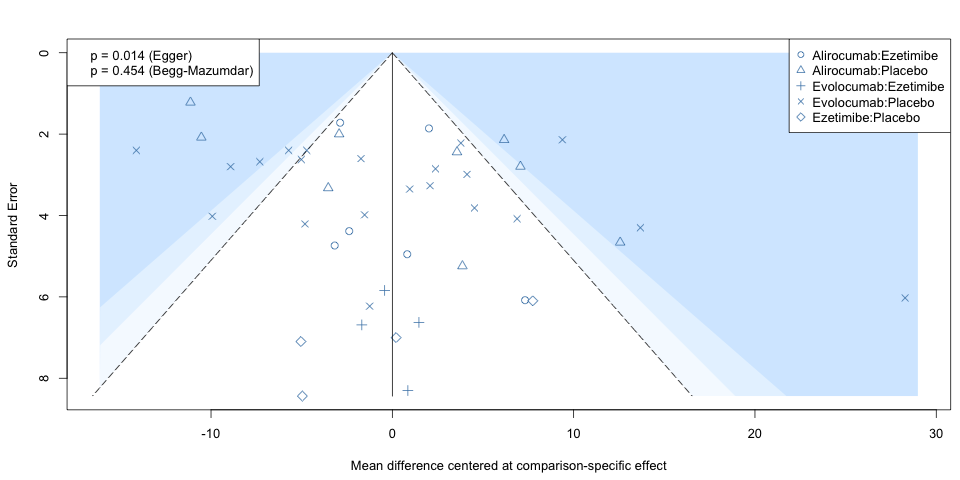


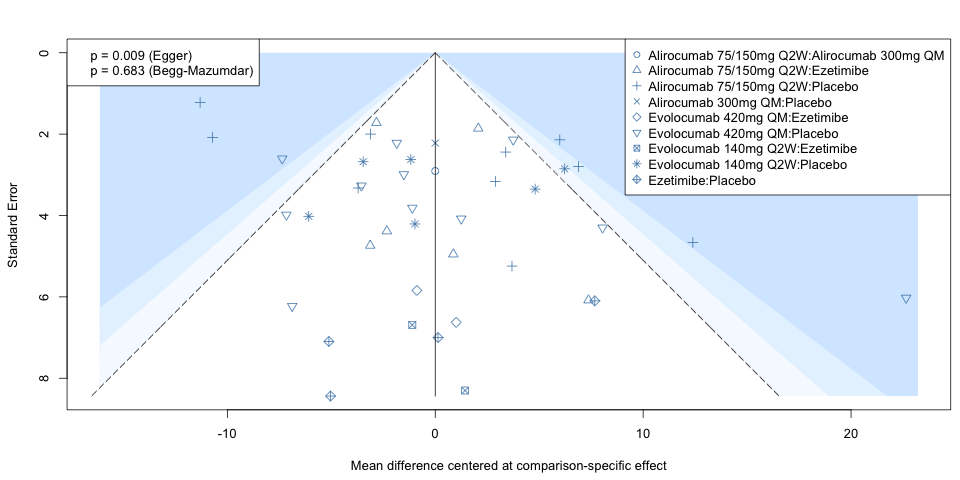


**Supplementary Figure 21. The assessment of publication bias for percentage change in lipoprotein(a)**

In funnel plot, each point represents a treatment contrast reported by a study. Asymmetry in the distribution of data points in a funnel plot may suggest publication bias. The dashed lines in funnel plot represent the 95% confidence interval given the standard error of the effect size, and 95% of the included studies should be within the triangular region. For network meta-analysis, reported effect sizes have been centered, so the solid vertical line is located at zero.


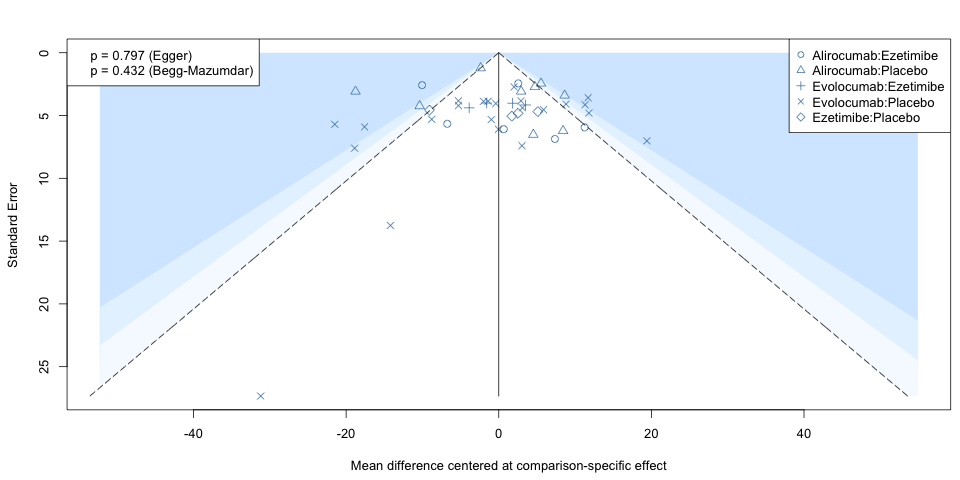


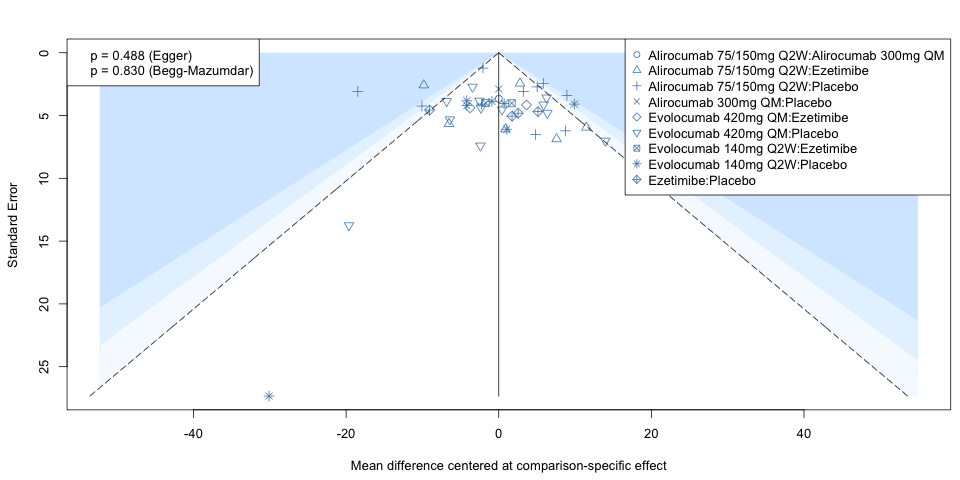


**Supplementary Figure 22. The assessment of publication bias for odds ratio of adverse event**

In funnel plot, each point represents a treatment contrast reported by a study. Asymmetry in the distribution of data points in a funnel plot may suggest publication bias. The dashed lines in funnel plot represent the 95% confidence interval given the standard error of the effect size, and 95% of the included studies should be within the triangular region. For network meta-analysis, reported effect sizes have been centered, so the solid vertical line is located at zero.


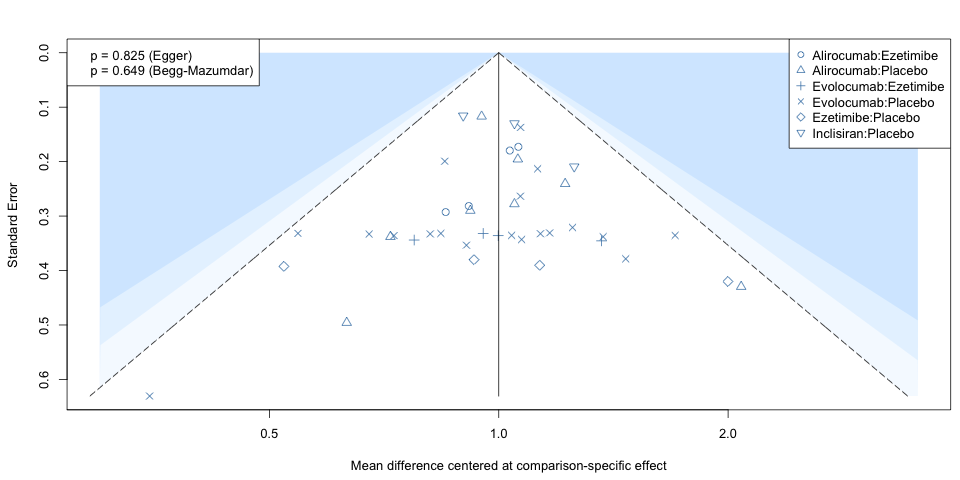


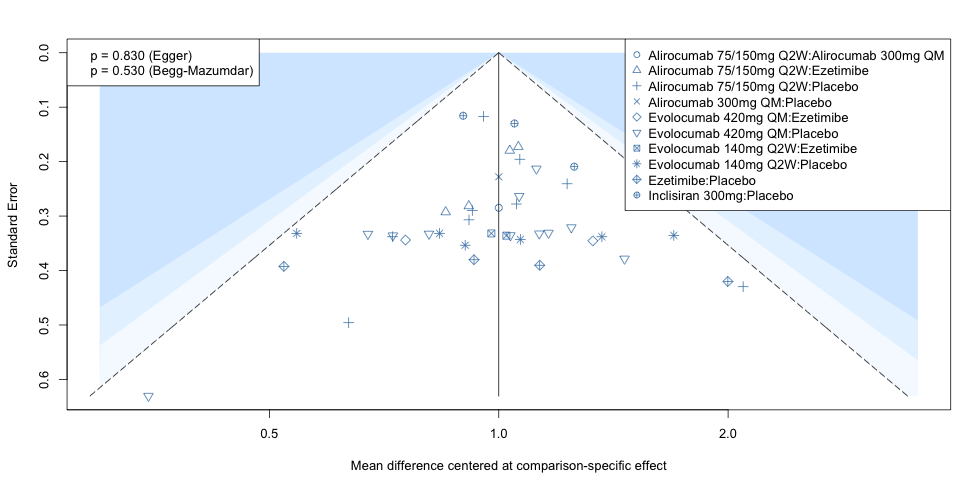


**Supplementary Figure 23. The assessment of publication bias for odds ratio of nasopharyngitis event**

In funnel plot, each point represents a treatment contrast reported by a study. Asymmetry in the distribution of data points in a funnel plot may suggest publication bias. The dashed lines in funnel plot represent the 95% confidence interval given the standard error of the effect size, and 95% of the included studies should be within the triangular region. For network meta-analysis, reported effect sizes have been centered, so the solid vertical line is located at zero.


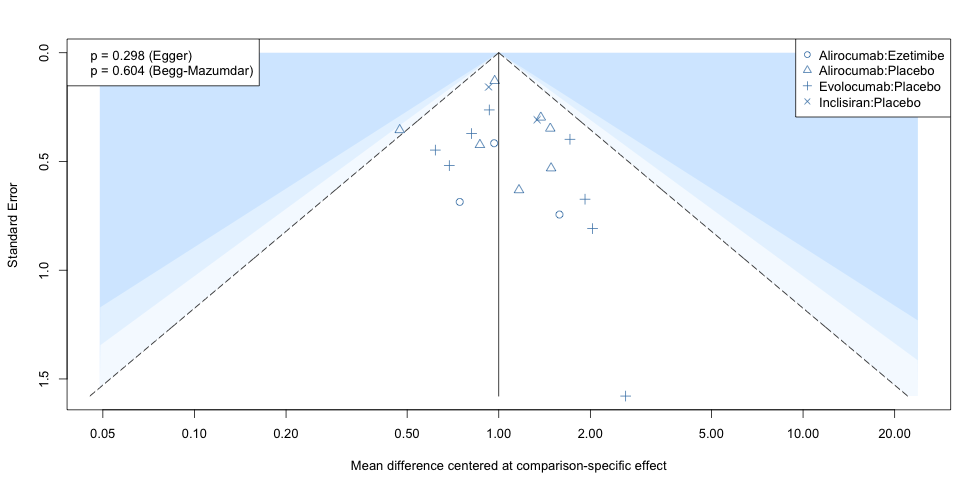


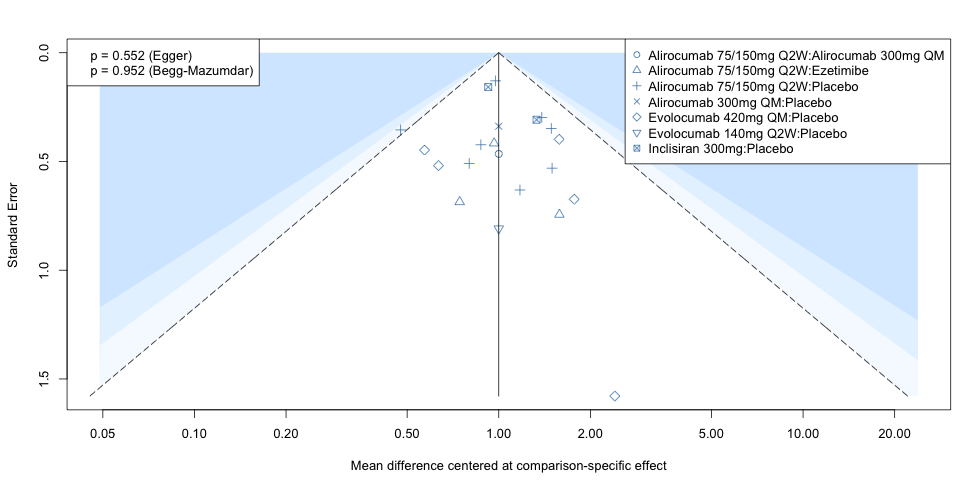


**Supplementary Figure 24. The assessment of publication bias for odds ratio of injection-site reaction**

In funnel plot, each point represents a treatment contrast reported by a study. Asymmetry in the distribution of data points in a funnel plot may suggest publication bias. The dashed lines in funnel plot represent the 95% confidence interval given the standard error of the effect size, and 95% of the included studies should be within the triangular region. For network meta-analysis, reported effect sizes have been centered, so the solid vertical line is located at zero.


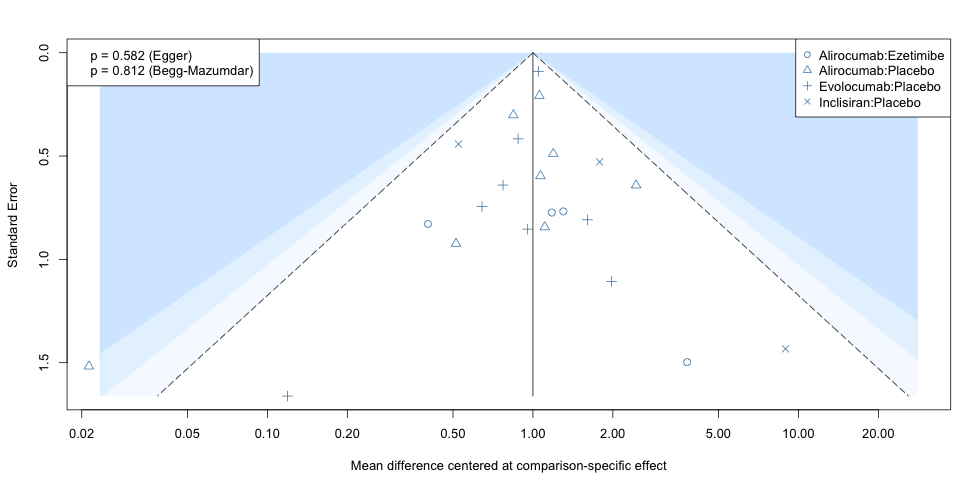


**
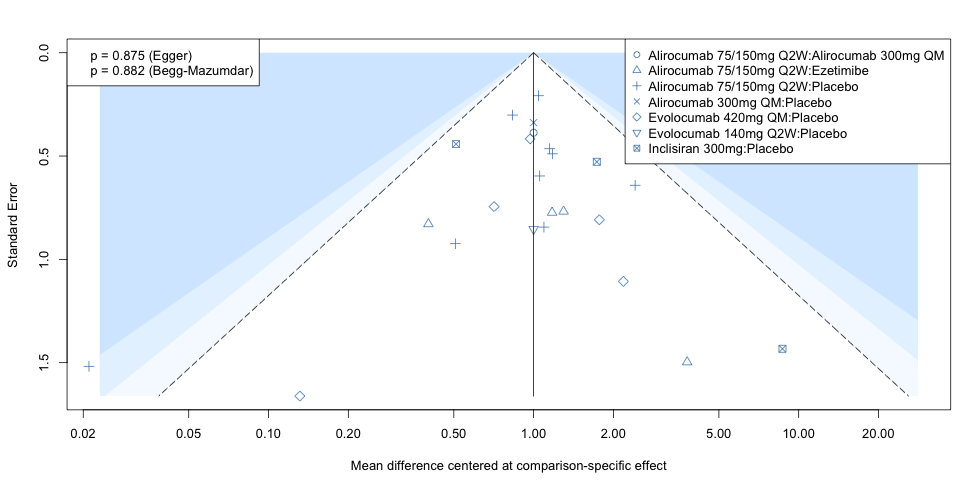
**

**Supplementary Figure 25. The assessment of publication bias for odds ratio of serious adverse event**

In funnel plot, each point represents a treatment contrast reported by a study. Asymmetry in the distribution of data points in a funnel plot may suggest publication bias. The dashed lines in funnel plot represent the 95% confidence interval given the standard error of the effect size, and 95% of the included studies should be within the triangular region. For network meta-analysis, reported effect sizes have been centered, so the solid vertical line is located at zero.


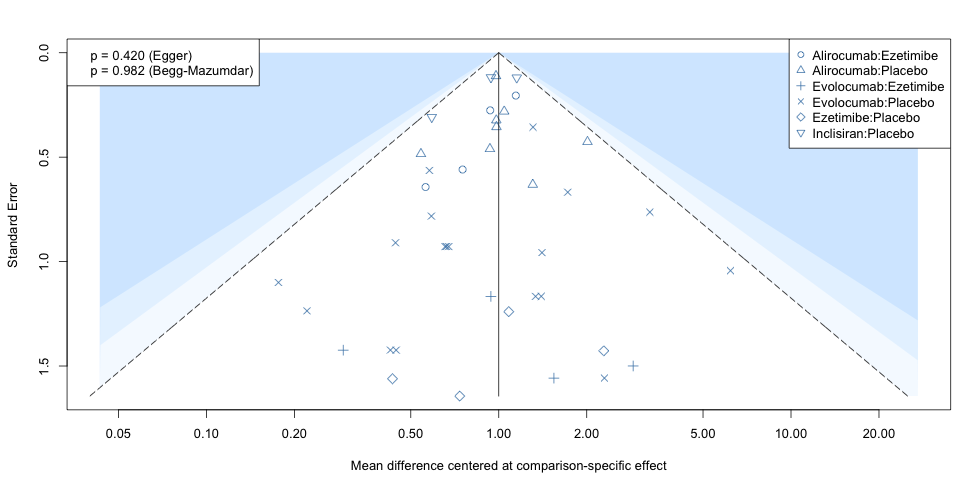


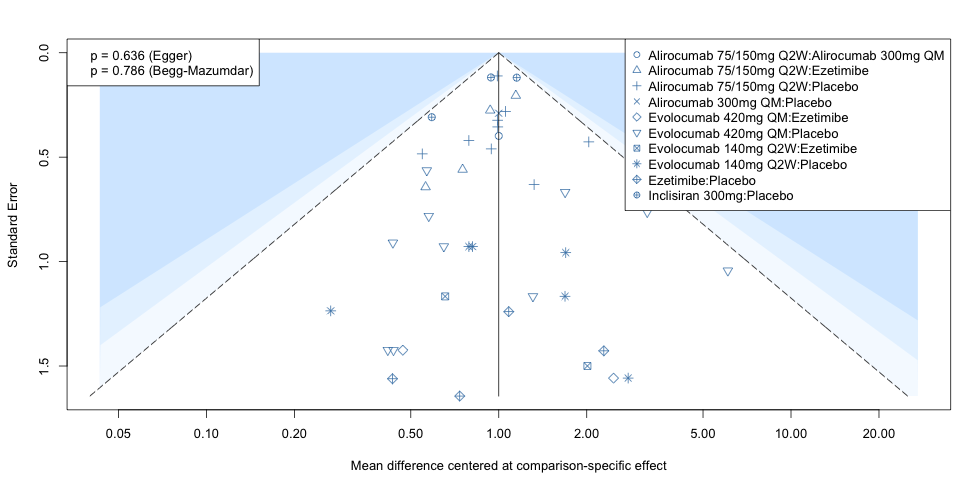


**Supplementary Figure 25. Sensitivity analysis of lipid change between PCSK9 inhibitors/ezetimibe and placebo using an** **alternative meta-analysis model, fixed-effect model**

**
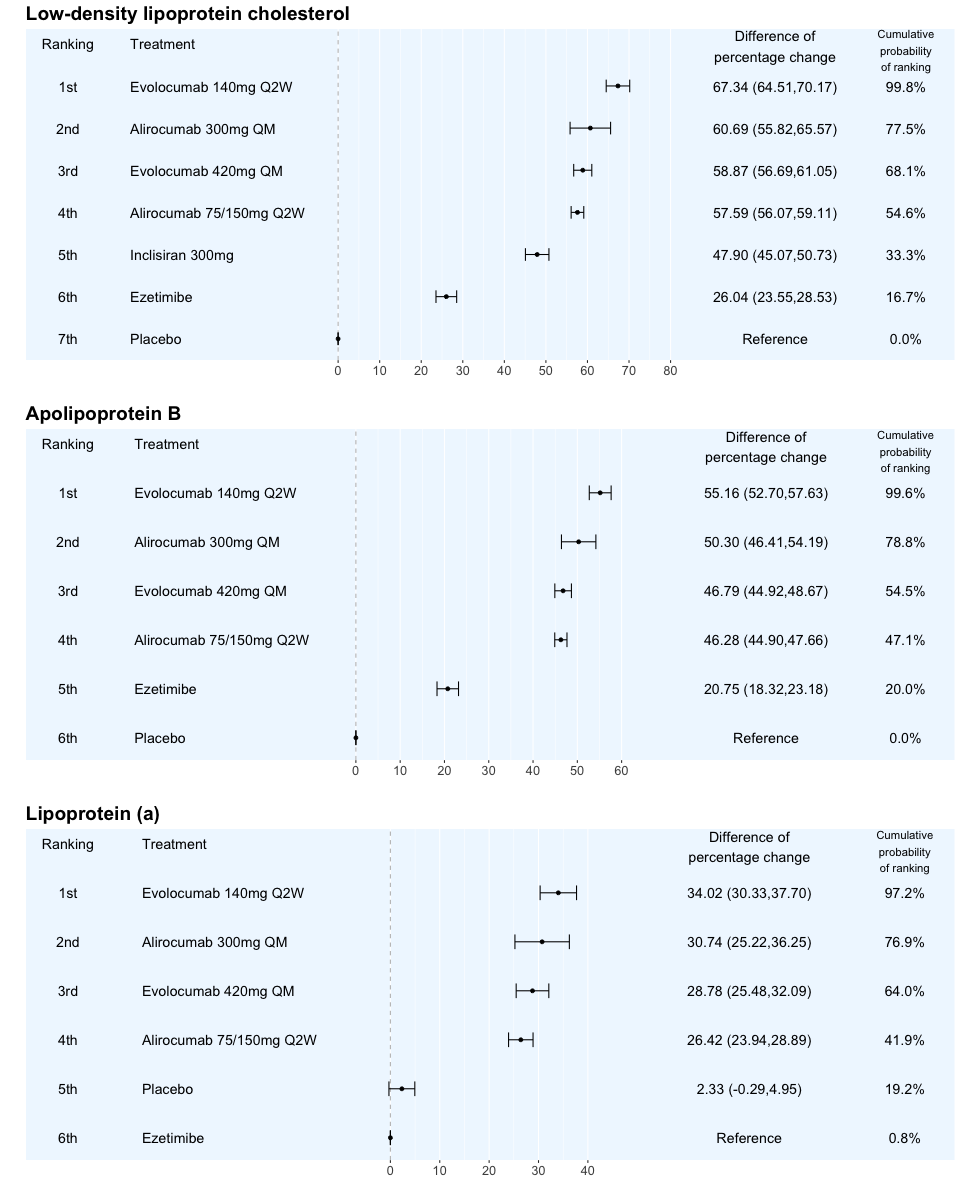
**

**Supplementary Figure 26.** **Sensitivity analysis of adverse event between PCSK9 inhibitors/ezetimibe and placebo using an alternative meta-analysis model, fixed-effect model**
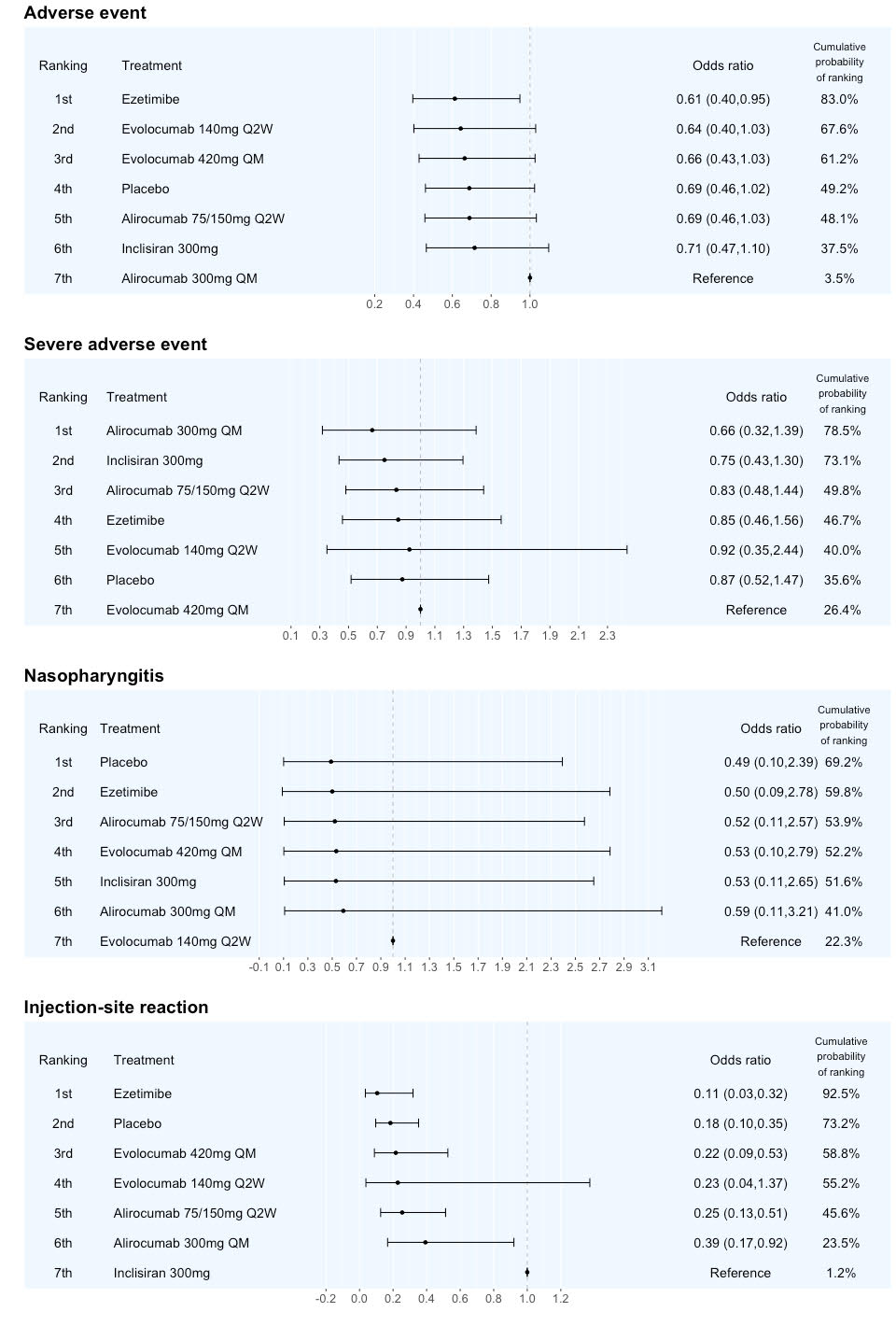


**Supplementary Table 1. Results of inconsistency testing**

| Outcomes | Within designs | Between designs | Between designs  (interaction random effect model) |
| --- | --- | --- | --- |
| Treatment |  |  |  |
| Low-density lipoprotein cholesterol | <0.001 | <0.001 | 0.103 |
| Apolipoprotein B | <0.001 | 0.119 | 0.588 |
| Lipoprotein(a) | <0.001 | 0.079 | 0.349 |
| Adverse events | 0.703 | 0.225 | 0.225 |
| Nasopharyngitis event | 0.720 | 0.535 | 0.535 |
| Infection-site reaction | 0.503 | - | - |
| Serious adverse events | 0.371 | - | - |
| Treatment with different drug dose |  |  |  |
| Low-density lipoprotein cholesterol | <0.001 | 0.002 | 0.414 |
| Apolipoprotein B | <0.001 | 0.005 | 0.629 |
| Lipoprotein(a) | <0.001 | 0.296 | 0.819 |
| Adverse events | 0.500 | 0.670 | 0.670 |
| Nasopharyngitis event | 0.703 | 0.733 | 0.733 |
| Infection-site reaction | 0.372 | 0.656 | 0.654 |
| Serious adverse events | 0.221 | 0.762 | 0.783 |

The table shows overall test for inconsistency models. If the p-value is not statistically significant, the overall inconsistency is low.

**Supplementary Table 2-1 The checklist of preferring reporting items for systematic reviews and meta-analyses**

| **Section/Topic** | **Item #** | **Checklist Item** | **Reported on Page #** |
| --- | --- | --- | --- |
| **TITLE** |  |  |  |
| Title | 1 | Identify the report as a systematic review | *Page 1* |
|  |  |  |  |
| **ABSTRACT** |  |  |  |
| Abstract | 2 | See the PRISMA 2020 for Abstracts checklist. | *Supplementary Table 2-2* |
|  |  |  |  |
| **INTRODUCTION** |  |  |  |
| Rationale | 3 | Describe the rationale for the review in the context of existing knowledge. | *Page 5-6* |
| Objectives | 4 | Provide an explicit statement of the objective(s) or question(s) the review addresses. | *Page 7-8* |
|  |  |  |  |
| **METHODS** |  |  |  |
| Eligibility criteria | 5 | Specify the inclusion and exclusion criteria for the review and how studies were grouped for the syntheses. | *Page 8* |
| Information sources | 6 | Specify all databases, registers, websites, organizations, reference lists and other sources searched or consulted to identify studies. Specify the date when each source was last searched or consulted. | *Page 8-9* |
| Search strategy | 7 | Present the full search strategies for all databases, registers, and websites, including any filters and limits used. | *Page 8*  *Supplementary Appendix 1* |
| Selection process | 8 | Specify the methods used to decide whether a study met the inclusion criteria of the review, including how many reviewers screened each record and each report retrieved, whether they worked independently, and if applicable, details of automation tools used in the process. | *Page 8*  *Supplementary Appendix 1* |
| Data collection process | 9 | Specify the methods used to collect data from reports, including how many reviewers collected data from each report, whether they worked independently, any processes for obtaining or confirming data from study investigators, and if applicable, details of automation tools used in the process. | *Page 8-9* |
| Data items | 10a | List and define all outcomes for which data were sought. Specify whether all results that were compatible with each outcome domain in each study were sought (e.g., for all measures, time points, analyses), and if not, the methods used to decide which results to collect. | *Page 9-10* |
|  | 10b | List and define all other variables for which data were sought (e.g., participant and intervention characteristics, funding sources). Describe any assumptions made about any missing or unclear information. | *Page 9* |
| Study risk of bias assessment | 11 | Specify the methods used to assess risk of bias in the included studies, including details of the tool(s) used, how many reviewers assessed each study and whether they worked independently, and if applicable, details of automation tools used in the process. | *Page 10* |
| Effect measures | 12 | Specify for each outcome the effect measure(s) (e.g., risk ratio, mean difference) used in the synthesis or presentation of results. | *Page 10-11* |
| Synthesis methods | 13a | Describe the processes used to decide which studies were eligible for each synthesis (e.g. tabulating the study intervention characteristics and comparing against the planned groups for each synthesis (item #5)). | *Page 10-11* |
|  | 13b | Describe any methods required to prepare the data for presentation or synthesis, such as handling of missing summary statistics, or data conversions. | *NA* |
|  | 13c | Describe any methods used to tabulate or visually display results of individual studies and syntheses. | *Page 11-12* |
|  | 13d | Describe any methods used to synthesize results and provide a rationale for the choice(s). If meta-analysis was performed, describe the model(s), method(s) to identify the presence and extent of statistical heterogeneity, and software package(s) used. | *Page 11-12* |
|  | 13e | Describe any methods used to explore possible causes of heterogeneity among study results (e.g. subgroup analysis, meta-regression). | *NA* |
|  | 13f | Describe any sensitivity analyses conducted to assess robustness of the synthesized results. | *Page 11* |
| Reporting bias assessment | 14 | Describe any methods used to assess risk of bias due to missing results in a synthesis (arising from reporting biases). | *Page 11* |
| Certainty assessment | 15 | Describe any methods used to assess certainty (or confidence) in the body of evidence for an outcome. | *Page 11* |
| **RESULTS†** |  |  |  |
| Study selection | 16a | Describe the results of the search and selection process, from the number of records identified in the search to the number of studies included in the review, ideally using a flow diagram. | *Page 12*  *Figure 1* |
|  | 16b | Cite studies that might appear to meet the inclusion criteria, but which were excluded, and explain why they were excluded. | *Page 12*  *Figure 1* |
| Study characteristics | 17 | Cite each included study and present its characteristics. | *Page 12-13*  *Table 1* |
| Risk of bias in studies | 18 | Present assessments of risk of bias for each included study. | *Supplementary Figure 1* |
| Results of individual studies | 19 | For all outcomes, present, for each study: (a) summary statistics for each group (where appropriate) and (b) an effect estimates and its precision (e.g. confidence/credible interval), ideally using structured tables or plots. | *NA* |
| Results of syntheses | 20a | For each synthesis, briefly summarise the characteristics and risk of bias among contributing studies. | *Supplementary Figure 2* |
|  | 20b | Present results of all statistical syntheses conducted. If meta-analysis was done, present for each the summary estimate and its precision (e.g. confidence/credible interval) and measures of statistical heterogeneity. If comparing groups, describe the direction of the effect. | *Page 13-16*  *Figure 3*  *Figure 4*  *Supplementary Figure 5*  *Supplementary Figure 6* |
|  | 20c | Present results of all investigations of possible causes of heterogeneity among study results. | *Page 16*  *Supplementary Table 1* |
|  | 20d | Present results of all sensitivity analyses conducted to assess the robustness of the synthesized results. | *Page 16* |
| Reporting biases | 21 | Present assessments of risk of bias due to missing results (arising from reporting biases) for each synthesis assessed. | *NA* |
| Certainty of evidence | 22 | Present assessments of certainty (or confidence) in the body of evidence for each outcome assessed. | *Figure 3-4*  *Supplementary Figure 5-18* |
|  |  |  |  |
| **DISCUSSION** |  |  |  |
| Discussion | 23a | Provide a general interpretation of the results in the context of other evidence. | *Page 16-17* |
|  | 23b | Discuss any limitations of the evidence included in the review. | *Page 19* |
|  | 23c | Discuss any limitations of the review processes used. | *Page 20* |
|  | 23d | Discuss implications of the results for practice, policy, and future research. | *Page 19-20* |
|  |  |  |  |
| **FUNDING** |  |  |  |
| Registration and protocol | 24a | Provide registration information for the review, including register name and registration number, or state that the review was not registered. | *Page 8*  *Supplementary Appendix 1* |
|  | 24b | Indicate where the review protocol can be accessed, or state that a protocol was not prepared. | *Supplementary Appendix 1* |
|  | 24c | Describe and explain any amendments to information provided at registration or in the protocol. | *Supplementary Appendix 1* |
| Support | 25 | Describe sources of financial or non-financial support for the review, and the role of the funders or sponsors in the review. | *Page 31* |
| Competing interests | 26 | Declare any competing interests of review authors. | *Page 31* |
| Availability of data, code and other materials | 27 | Report which of the following are publicly available and where they can be found: template data collection forms; data extracted from included studies; data used for all analyses; analytic code; any other materials used in the review. | *Table 1*  *Supplementary Appendix 1* |

**Supplementary Table 2-2** **PRISMA 2020 for abstracts checklist**

| **Section/Topic** | **Item #** | **Checklist Item** | **Reported (Yes/No)** |
| --- | --- | --- | --- |
| **TITLE** |  |  |  |
| Title | 1 | Identify the report as a systematic review | Yes |
|  |  |  |  |
| **BACKGROUND** |  |  |  |
| Objectives | 2 | Provide an explicit statement of the main objective(s) or question(s) the review addresses. | Yes |
|  |  |  |  |
| **METHODS** |  |  |  |
| Eligibility criteria | 3 | Specify the inclusion and exclusion criteria for the review. | Yes |
| Information sources | 4 | Specify the information sources (e.g. databases, registers) used to identify studies and the date when each was last searched. | Yes |
| Risk of bias | 5 | Specify the methods used to assess risk of bias in the included studies. | No |
| Synthesis of results | 6 | Specify the methods used to present and synthesise results. | Yes |
| **RESULTS†** |  |  |  |
| Included studies | 7 | Give the total number of included studies and participants and summarise relevant characteristics of studies. | Yes |
| Synthesis of results | 8 | Present results for main outcomes, preferably indicating the number of included studies and participants for each. If meta-analysis was done, report the summary estimate and confidence/credible interval. If comparing groups, indicate the direction of the effect (i.e. which group is favoured). | Yes |
|  |  |  |  |
| **DISCUSSION** |  |  |  |
| Limitations of evidence | 9 | Provide a brief summary of the limitations of the evidence included in the review (e.g. study risk of bias, inconsistency and imprecision). | No |
| Interpretation | 10 | Provide a general interpretation of the results and important implications. | Yes |
|  |  |  |  |
| **OTHER** |  |  |  |
| Funding | 11 | Specify the primary source of funding for the review. | No |
| Registration | 12 | Provide the register name and registration number. | No |
